# Single-cell transcriptomic profiling reveals innate-like cytotoxic intraepithelial lymphocyte expansion during *Salmonella* Enteritidis infection in chickens

**DOI:** 10.3389/fimmu.2026.1842283

**Published:** 2026-06-03

**Authors:** Shuja Majeed, Bikas Raj Shah, Bikash Aryal, Nimra Khalid, Tae Hyun Kim, Ali Nazmi

**Affiliations:** 1 Department of Animal Sciences, College of Food, Agricultural, and Environmental Sciences, The Ohio State University, Wooster, OH, United States; 2 Department of Animal Science, College of Agricultural Sciences, The Pennsylvania State University, University Park, PA, United States; 3 Food For Health Discovery Theme, The Ohio State University, Columbus, OH, United States

**Keywords:** chicken, intraepithelial lymphocytes, layer, *Salmonella*, ScRNA-seq

## Abstract

*Salmonella* is a major foodborne pathogen that causes approximately 1.35 million infections annually in the US and remains a leading cause of poultry-associated foodborne illness. To improve chickens’ resistance to this pathogen, it is important to understand the mucosal immune mechanisms that govern intestinal defense. Intraepithelial lymphocytes (IELs) positioned between intestinal epithelial cells provide frontline immune surveillance against enteric pathogens. However, a comprehensive characterization of IEL subtype responses to *Salmonella* infection remains incomplete. Therefore, we conducted this study to examine IEL subtypes and their mechanisms in response to a *Salmonella* enterica serovar Enteritidis (*S.* Enteritidis) challenge using a combination of spectral flow cytometry and single-cell RNA sequencing (scRNA-seq). Fifty specific-pathogen free (SPF) chicks were reared to 21 days of age and then assigned to *S.* Enteritidis-challenged (SE; 1.62 × 10^8^ CFU/bird, oral gavage) or control (CN; PBS) groups (n = 25/group). On day 2 post infection (2 dpi) and 6 dpi, eight birds per group were sampled to collect liver and ceca for bacteriology and ileum for IEL acquisition. Bacteriological findings confirmed the challenge: the SE group harbored *S.* Enteritidis at both time points. Flow cytometry results showed that *Salmonella* challenge increased the proportion of TCRγδ^+^CD8αβ^+^ cytotoxic IELs at 2 dpi, as well as the overall IEL proportion at 2 and 6 dpi. Notably, scRNA-seq identified clusters of progenitor T cells that significantly expanded and innate-like cytotoxic T cells, which emerged in SE-challenged birds at 2 dpi, indicating rapid mobilization of an innate-like cytotoxic response. Integration of flow cytometry and scRNA-seq data provided evidence that cytotoxic T cells expressing CD8αβ acquire innate-like transcriptional signatures within the intestinal epithelial compartment, suggesting functional reprogramming that enables rapid antigen responses. Trajectory analysis identified a robust transcriptionally inferred trajectory from progenitor T cells through activated CD8^+^ T cells to innate-like CTL as the predicted terminal cluster, with quiescent stem-like resident memory T cells transcriptionally positioned as a reservoir. These findings reveal a previously uncharacterized innate-like cytotoxic IEL response as a critical early defense mechanism against *Salmonella* in poultry and identify self-renewing stem-like Trm cells as a reservoir for rapid IEL effector differentiation.

## 1 Introduction

Intraepithelial lymphocytes (IELs) reside within the intestinal epithelium, where they serve as sentinels at the interface between the host and the luminal environment (1). In mammals, IELs exhibit remarkable heterogeneity, encompassing conventional TCRαβ^+^ and TCRγδ^+^ T cells, innate-like lymphocytes, and populations with hybrid phenotypes that bridge adaptive and innate immunity (1, 2). Recent single-cell transcriptomic studies in humans and mice have revealed that IELs can undergo functional reprogramming within the epithelial niche, with conventional CD8αβ^+^ T cells acquiring innate-like cytotoxic capacity in response to epithelial stress signals (2). However, whether similar IEL plasticity and innate-like reprogramming exist in chickens, and how IEL subsets respond to enteric pathogens at the transcriptional level, remains unknown. Therefore, characterizing these mechanisms in chickens is of both biological and practical significance.

*Salmonella* enterica, particularly serovars Enteritidis and Typhimurium, is a leading cause of foodborne zoonotic disease, with approximately 1.35 million infections annually in the United States, of which poultry consumption accounts for over 23% (3). Chickens commonly harbor *Salmonella* subclinically, acting as asymptomatic reservoirs that transmit the pathogen into the human food chain (4). Numerous advances in poultry genetics, husbandry, and nutrition have substantially increased both egg and meat production to meet the strong demand. However, disease challenges are a constant issue. Moreover, with increasing restrictions on prophylactic antibiotic use, strategies to enhance the birds’ mucosal immune resistance could reduce *Salmonella* carriage at the source, but such strategies require a detailed understanding of the intestinal immune cells that mediate early defense (5, 6).

Previous studies in chickens have established that IELs respond to a range of enteric pathogens, including *Eimeria acervulina* (7, 8), Newcastle disease virus (9), infectious bursal disease virus (10, 11), and bacterial infections (12, 13). Our group previously characterized IEL subset dynamics following *Salmonella* Typhimurium and *Clostridium perfringens* challenge using flow cytometry, demonstrating expansion of cytotoxic and natural IEL populations (14, 15). However, flow cytometry is constrained by the limited availability of chicken-specific antibodies and cannot resolve the transcriptional heterogeneity or functional states of individual IEL subsets.

To address these limitations, we performed the first single-cell RNA sequencing (scRNA-seq) analysis of chicken IELs following *Salmonella* Enteritidis challenge, complemented by spectral flow cytometry. This approach enabled us to identify novel IEL populations and to understand their transcriptional programs at the cellular level, revealing dynamic shifts in the IEL compartment that were previously undetectable with conventional methods.

## 2 Materials and methods

### 2.1 Ethics statement

The Ohio State University (OSU)’s Institutional Animal Care and Use Committee (2019A00000118-R1) approved this study, and it was conducted in compliance with all relevant guidelines and regulations.

### 2.2 Experimental design

**Figure 1:**
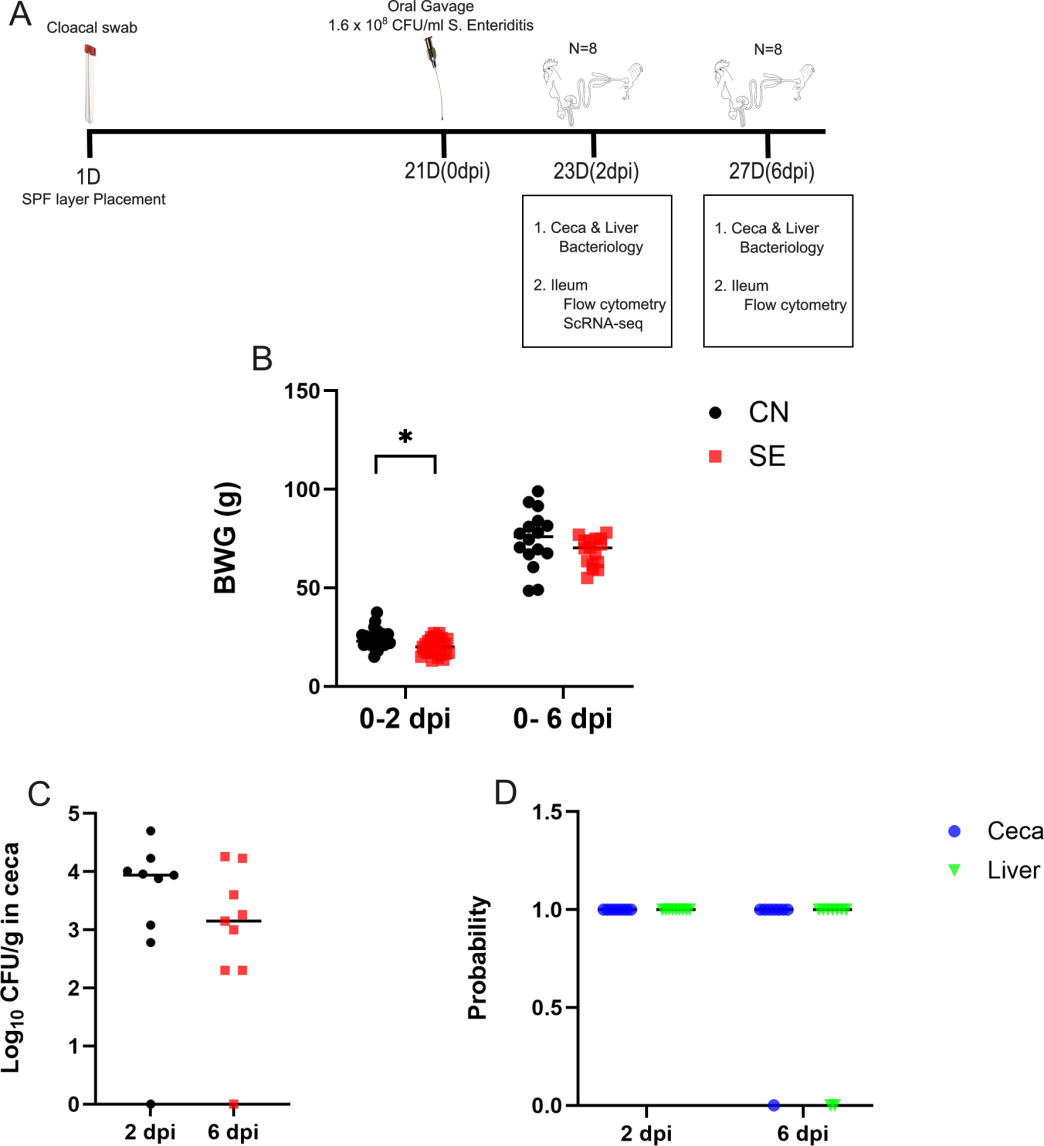
Schematic overview of the experimental design along with performance and bacteriological assessment of SPF layer chickens following oral challenge with *Salmonella* Enteritidis. **(A)** SPF layer chicks were placed on day 1 of age, and cloacal swabs were collected to confirm *Salmonella* negative status. On day 21 (0 dpi), birds were orally gavaged with 1.62 × 10^8^ CFU/bird of *S.* Enteritidis (challenge group, SE) or PBS (control group, CN) (n = 25/group). At 2 dpi (day 23) and 6 dpi (day 27), eight birds per group were euthanized for sample collection. Ceca and liver were collected for bacteriological analysis, and the ileum was harvested for IEL isolation. Isolated IELs were enriched for CD45^+^ leukocytes by magnetic-activated cell sorting (MACS) and subsequently used for spectral flow cytometry and single-cell RNA sequencing (scRNA-seq). Body weights were recorded at 0, 2, and 6 dpi. scRNA-seq was performed at 2 dpi only. **(B)** Body weight gain (BWG, g) of CN and SE groups measured over two intervals: 0–2 days post infection (dpi) and 0–6 dpi. Each point represents an individual bird; horizontal bars indicate the group mean. **(C)** Cecal S. Enteritidis counts determined by direct plating, expressed as log_10_ CFU/g, at 2 and 6 dpi. Each point represents an individual bird; horizontal bars indicate the group mean (n = 8 per group). **(D)** Probability of S. Enteritidis recovery from ceca and liver following enrichment culture at 2 and 6 dpi. Each point represents an individual bird (probability = 1 indicates Salmonella-positive culture, probability = 0 indicates negative culture). Asterisk (*) indicates a statistically significant difference between groups (*P* < 0.05).

Fifty SPF layer birds were placed on the day of hatch on sawdust litter in an environmentally controlled house (**Figure 1A**). SPF birds were obtained from the Center for Food Animal Health, The Ohio State University. These birds are white leghorn chickens from a flock routinely monitored and certified as SPF according to CFAH health monitoring testing protocol by the OSU IACUC and Institutional Biosafety Committees (IBCs). On day 1 of age, 20 birds were selected at random from the 50 incoming SPF chicks by sequential capture from the housing pen, without operator preference or systematic ordering, for cloacal swab collection to confirm *Salmonella*-negative flock status prior to challenge. Afterward, the birds were divided into two equal groups of 25: Control (CN) and *Salmonella* Enteritidis challenged (SE). On day 21 of age, the CN group was mock challenged with PBS, while the SE group was orally gavaged with 1.62 × 10^8^ CFU/ml of *Salmonella* Enteritidis P13a (*S.* Enteritidis). Eight birds per treatment were euthanized through CO_2_ asphyxiation (CO_2_ was gradually introduced at a controlled flow rate, displacing approximately 45% of the chamber volume per minute, following cessation of respiration, death was confirmed by removing vital organs) on day 23 [2 days post infection (2 dpi)] and day 27 (6 dpi) of age. At each time point, liver and ceca were collected for bacteriology, and ileum (approximately 10 cm section) was sampled for isolation of IELs. On 0 dpi (challenge day), 2 dpi, and 6 dpi, body weight was also recorded to assess live performance.

### 2.3 *Salmonella* challenge dose preparation

*Salmonella* Enteritidis (P13a) stock solution was retrieved from a -80 °C freezer and propagated in Tryptic Soy Broth (Sigma-Aldrich, St. Louis, MO, USA) overnight and subsequently passaged twice to reach logarithmic phase. Bacteria were washed, resuspended in PBS, and adjusted to approximately 1 × 10^8^ CFU/ml utilizing a spectrophotometer at a 625 nm wavelength. Concentration was confirmed by plating on XLT4 agar (Sigma-Aldrich, USA) supplemented with novobiocin (20 μg/ml) and nalidixic acid (20 μg/ml). The final challenge dose was 1.62 × 10^8^ CFU/ml administered at 1 ml per bird via oral gavage.

### 2.4 *Salmonella* recovery and enumeration

On day 1 of age, cloacal swabs were collected from 20 birds, incubated overnight in tetrathionate broth at 37 °C, and plated on XLT4 agar to confirm *Salmonella*-negative status prior to challenge. At 2 dpi and 6 dpi, liver and ceca were collected in sterile, filtered bags (Whirl-Pak, Pleasant Prairie, WI, USA). Each sample was weighed, diluted with PBS to two times the tissue weight, and mechanically homogenized. Homogenates were serially diluted and plated on XLT4 agar supplemented with novobiocin (20 μg/ml) and nalidixic acid (20 μg/ml). Bacterial colonies were enumerated to obtain CFU/g and assess the recovery of *S.* Enteritidis from the birds. Negative samples were enriched overnight in tetrathionate broth at 37 °C and re-plated on XLT-4 agar (37 °C, 24 h) for qualitative assessment.

### 2.5 IELs isolation and CD45^+^ enrichment

IELs were isolated from ileum as previously described (14). Briefly, the ileal tissues were flushed with PBS and opened longitudinally, the mucosa gently scraped off, resuspended in PBS supplemented with 5% chicken serum, 2 mM EDTA, and 2 mM DTT, and then placed on a shaking incubator (37 °C, 150 rpm) for 45 min. Following incubation, the supernatant was passed through a gauze column, resuspended in 40% Percoll (Cytiva, Marlborough, MA, USA), and overlaid onto a 70% Percoll for density gradient centrifugation. The IEL fractions from 8 individual birds were pooled to yield four samples per treatment. Subsequently, pooled samples were filtered through 40 μm cell strainers and enriched for CD45^+^ (Leukocytes) cells using Magnetic-Activated Cell Sorting (MACS) using a CD45-biotin conjugated antibody (Southern Biotech, Birmingham, AL, USA) on MS columns (Miltenyi Biotec, Bergisch Gladbach, Germany). Following MACS, enriched IELs were quantified by the trypan blue method and divided for flow cytometry and scRNA-seq.

### 2.6 Flow cytometry

For flow cytometry, approximately 1 × 10^6^ enriched IELs per sample were stained with fluorochrome-conjugated anti-chicken CD45 SPRD (LT40), CD4 PE-CY7 (CT-4), CD3 AF647 (CT-3), TCRγδ FITC (TCR-1), CD8α AF700 (CT-8), and CD8β PE (EP42) antibodies (Southern Biotech, USA). Ghost viability dye Red 510 (Tonbo Biosciences, San Diego, CA, USA) was used to exclude dead cells. Stained cells were acquired on a Cytek Northern Lights flow cytometer (Cytek, Fremont, CA, USA). FlowJo v10.8.1 (BD Biosciences, USA) was used to analyze cell frequencies, and the gating strategy was consistent with our previous study (14). Cell distributions were expressed as percentages of the parent population.

### 2.7 ScRNA-seq, library preparation and sequencing

For scRNA-seq, 4 pooled samples (each representing 2 birds) at 2 dpi were further pooled in pairs, yielding 2 scRNA-seq samples per group (**Supplementary Figure 1**). Pooled samples were then shipped to the OSU Single Cell Omics Core where cells were washed and resuspended in PBS + 5% FBS solution to remove residual cell culture media. Approximately 5000–13000 cells per sample were loaded onto the Chromium X instrument (10x Genomics, Pleasanton, CA, USA) using the GEM-X Universal 3’ Gene Expression v4 kit (10x Genomics), which partitioned cells into Gel Beads-in-Emulsion (GEMs) for barcoding and cDNA synthesis. Four sequencing libraries (two libraries/group) were constructed using Library Construction Kit C (10x Genomics) and sequenced at Admera Health in South Plainfield, NJ, on a NovaSeq X Plus instrument (Illumina, San Diego, CA), generating 2 × 150 bp paired-end reads at an average depth of 70,000 reads/cell. FASTQ files received post-sequencing were processed using the Cell Ranger v9.0.1 pipeline (10x Genomics) to align reads, quantify gene expression, and generate count matrices for downstream analysis. Reads were aligned to the chicken reference genome (NCBI taxa: 9031. Assembly: RefSeq GCF_016699485.2; GenBank GCA_016699485.1).

### 2.8 ScRNA-seq filtering, normalization, and cluster generation

Count matrices were loaded into R v 4.4.2 as a Seurat v5.0 (16) object. Ambient RNA contamination was removed using SoupX v1.6.2 (17). Cells with mitochondrial percentages greater than 20%, feature counts less than 300, and features present in fewer than 3 cells were filtered out. Potential doublets were also removed by excluding cells with more than 6000 features and through DoubletFinder v2.0.4 (18) package. Following quality control, each Seurat object was normalized using SCTransform(). The four normalized Seurat objects were integrated using PrepSCTIntegration() and SelectIntegrationFeatures() based on 3,000 variable features. Anchor cells were defined using FindIntegrationAnchors() with normalization method set to “SCT”. The anchors were integrated using IntegrateData() with the normalization method set to “SCT”, yielding a combined Seurat object for further analysis. Dimensionality reduction was performed using principal component analysis (PCA), and the optimal number of principal components was determined using an elbow plot. The first seven principal components were used for graph-based clustering with FindNeighbors() and FindClusters() (resolution = 0.2), and UMAP was applied for visualization. Resolution was optimized by using the R package clustree v0.5.1 (19) (**Supplementary Figure 2**).

### 2.9 Cluster annotation, proportions, and gene expression

Cluster identities were manually annotated using the top 50 differentially expressed genes (DEGs) per cluster (identified via FindMarkers()) and the canonical marker gene expression using published chicken, human and mouse single-cell references. DEGs between the CN and SE groups within each cluster were determined using the R package MAST v1.33.0 (20), with counts per million (CPM) normalization and Cellular Detection Rate (CDR) as a covariate. Genes with |log_2_FC| > 0.5 and adjusted p value < 0.05 were considered statistically significant. Differences in cell proportions between groups were assessed using propeller function from the speckle v1.8.0 R package (21). This method applies a logit transformation to the cell-type proportions and tests for differences using a linear modeling framework (*limma*). Clusters with adjusted *P* < 0.05 were considered statistically significant.

### 2.10 Functional enrichment analysis

Gene Ontology (GO) and Kyoto Encyclopedia of Genes and Genomes (KEGG) enrichment analysis were performed to interpret transcriptomic differences between the CN and SE groups for each annotated cluster. Over-representation analysis (ORA) was applied via ClusterProfiler v4.16.0 (22). Differentially expressed genes with *P* < 0.05 were used as input, with all genes expressed in the dataset as the background gene universe. Enrichment significance was evaluated using a hypergeometric test, and p-values were corrected for multiple comparisons using the Benjamini–Hochberg FDR method. Pathways with an FDR-adjusted p-value < 0.05 were considered significantly enriched.

### 2.11 Trajectory inference

Pseudotime trajectory analysis was performed using Slingshot package v2.8.0 (23). The Seurat object was converted to a SingleCellExperiment object, with UMAP coordinates used as the reduced dimensionality input. Trajectory inference was performed using slingshot() with clusterLabels set to the Seurat cluster identities. Lineage topology was extracted using slingLineages() and pseudotime values were obtained using slingPseudotime(). Furthermore, differential expression analysis along pseudotime was performed using the tradeSeq package v1.14.0 (24).

### 2.12 Statistical analysis

GraphPad Prism v10.6.0 (GraphPad, Boston, MA, USA) was used for statistical analysis. Bacteriology and body weight gain data were analyzed using Student’s t-test. Cell frequencies acquired through flow cytometry were compared between groups using the nonparametric Mann–Whitney U test. P value < 0.05 was considered statistically significant.

## 3 Results

### 3.1 *S.* Enteritidis challenge established a subclinical infection model

Body weight gain (BWG) was calculated for 0–2 dpi and 0–6 dpi (**Figure 1B**). The SE group exhibited a significant reduction in BWG at 2 dpi (*P* < 0.05), which recovered by 6 dpi as demonstrated by similar BWG between the groups. No clinical signs or mortality were observed during the trial. Cecal bacteriology confirmed that the infected group (SE) harbored *S.* Enteritidis, whereas the CN group was negative. At 2 dpi, the SE group was colonized by 4.21 × 10^3^ CFU/g of *S.* Enteritidis, which reduced to 1.99 × 10^3^ CFU/g at 6 dpi (**Figure 1C**). In liver, direct plating yielded no *Salmonella* contamination; however, after enrichment, 88% were positive for *S.* Enteritidis at 2 dpi and 78% at 6 dpi (**Figure 1D**). Together, these results confirm a subclinical *S.* Enteritidis infection, consistent with the carrier state commonly observed in chickens (25–27).

### 3.2 *S.* Enteritidis challenge increased overall IEL and TCRγδ^+^CD8αβ^+^ IEL proportions at 2 dpi

**Figure 2:**
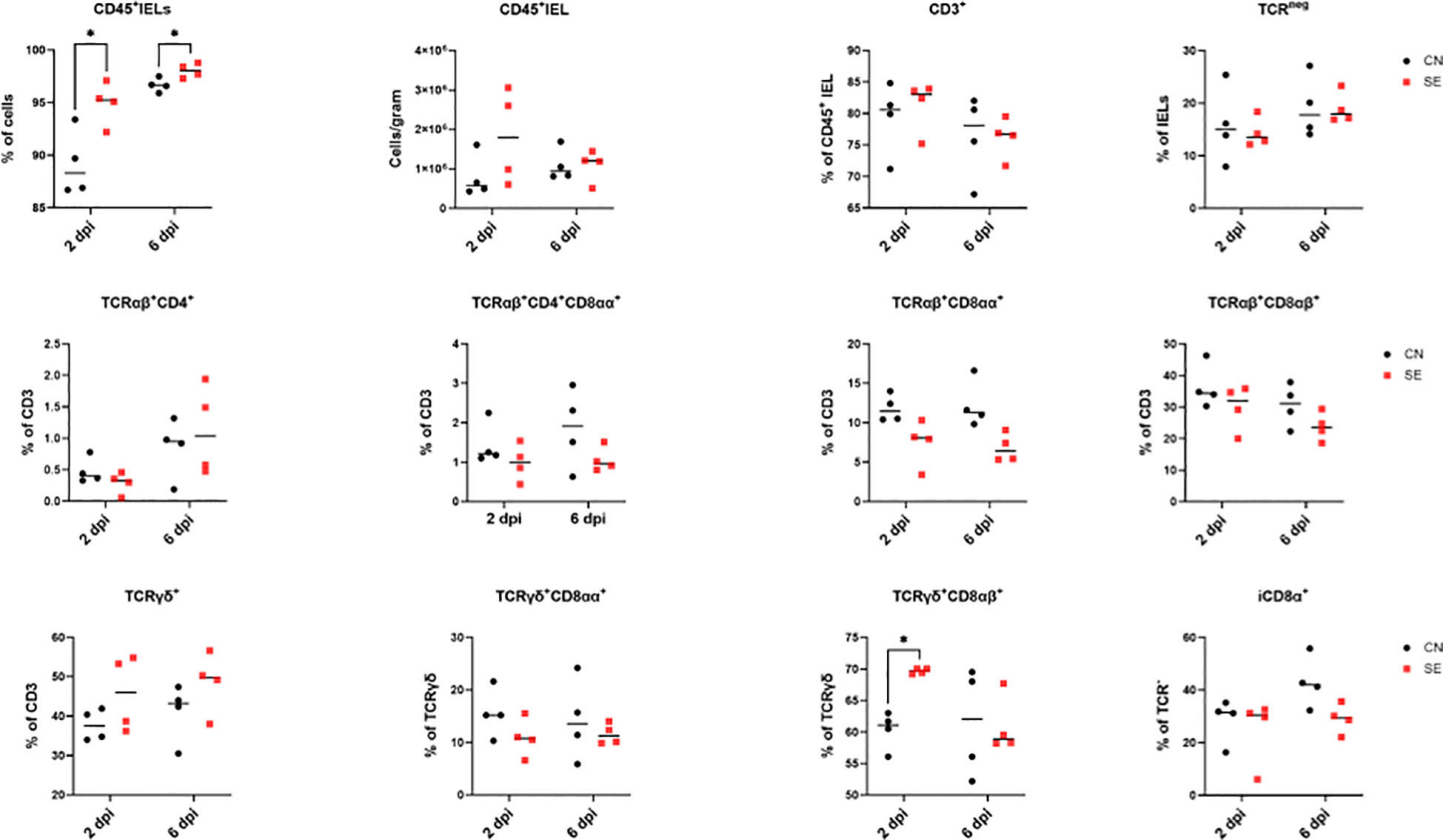
Effect of *Salmonella* Enteritidis challenge on ileal intraepithelial lymphocyte (IEL) subpopulations at 2 and 6 days post infection (dpi). Twenty-one-day-old SPF layer chickens were orally gavaged with 1.62 × 10^8^ CFU/bird of *S.* Enteritidis (challenge group, SE) or PBS (control group, CN). Ileal IELs were isolated and phenotyped by spectral flow cytometry (n = 4 pooled samples per group per time point). Panels show CD45^+^ IEL proportion and absolute count, TCRαβ^+^ subsets (CD4^+^, CD4^+^CD8αα^+^, CD8αα^+^, CD8αβ^+^), TCRγδ^+^ subsets (total, CD8αα^+^, CD8αβ^+^), TCR^-^ cells, CD3^+^ cells, and iCD8α cells at 2 and 6 dpi. Each point represents one biological replicate (one pool of birds); horizontal bars indicate the group mean. Statistical differences between CN and SE groups were determined using the Mann–Whitney U test (* *P* < 0.05).

MACS-based CD45^+^ enrichment increased leukocyte purity from approximately 20% to 73%(**Supplementary Figure 3**), after which each sample was split into two aliquots for downstream analysis. Flow cytometric analysis of ileal IELs revealed a comprehensive panel of IEL subpopulations at 2 and 6 dpi (**Figure 2**). The frequency of CD45^+^ IELs was significantly higher in the SE group at both 2 dpi and 6 dpi (*P* < 0.05). Among the IEL subsets examined, only TCRγδ^+^CD8αβ^+^ IELs represented a significantly higher percentage in the SE group at 2 dpi (*P* < 0.05). However, by 6 dpi, this difference was no longer observed, and the rest of the IEL subtypes, including TCRαβ^+^CD8αβ^+^, TCRαβ^+^CD4^+^, TCRαβ^+^CD8αα^+^, TCRγδ^+^CD8αα^+^, TCR^-^, and innate CD8α (iCD8α^+^) populations, showed similar distributions between the CN and SE groups at both time points. The selective increase in TCRγδ^+^CD8αβ^+^ IELs indicated an early shift towards cytotoxicity within the γδ T cell compartment to counter the *Salmonella* challenge, as CD8αβ is a canonical co−receptor expressed on classical MHCI−restricted cytotoxic T lymphocytes and is routinely used as a marker to identify cytotoxic CD8^+^ T cells (28).

### 3.3 Single-cell RNA sequencing output

Cell Ranger output reported an average sequencing saturation of 94%, fraction reads in cells of 80%, 21,395 gene detections, and a median UMI count of 2,661 per cell across all samples. After quality control and filtering, a total of 31,687 cells were recovered across all four samples, with a median of 1143 genes detected per cell.

### 3.4 Distinct cell lineages identified following clustering in the ileal epithelial compartment

**Figure 3:**
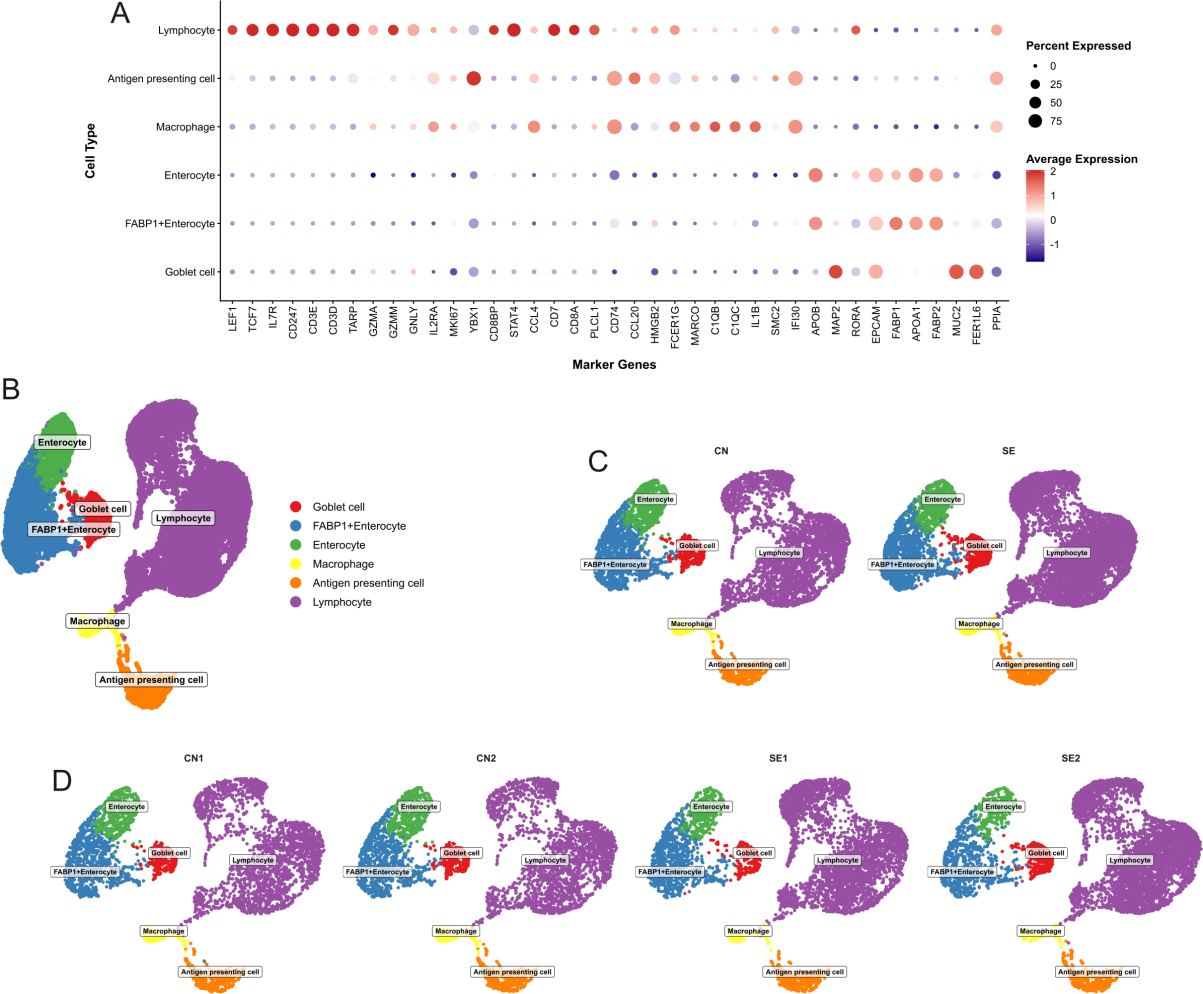
Global single-cell RNA sequencing clustering of ileal intraepithelial cells from *S.* Enteritidis-challenged and control chickens at 2 days post infection (dpi). Twenty-one-day-old SPF layer chickens were orally gavaged with 1.62 × 10^8^ CFU/bird of *S.* Enteritidis (challenge group, SE) or PBS (control group, CN). IELs isolated from the ileum at 2 dpi were processed using the 10x Genomics Chromium platform (n = 2 pooled samples per group) and analyzed using the Seurat pipeline. **(A)** Dot plot showing marker gene expression across identified cell types. Dot size represents the percentage of cells expressing each gene, and color intensity indicates the average expression level. **(B)** UMAP visualization of all identified cell types across both groups: FABP1^+^ enterocytes, goblet cells, antigen-presenting cells, lymphocytes, enterocytes, and macrophages. **(C)** UMAP plots split by treatment group (CN and SE). **(D)** UMAP plots split by individual replicates (CN1, CN2, SE1, and SE2).

Cells were identified based on the top 50 differentially expressed genes (DEGs) between clusters, along with the expression of canonical marker genes (**Figure 3A**; **Supplementary Data Sheet 1**). Initial clustering revealed six major cell populations within the ileal epithelial compartment, including lymphocytes (60.3% of total cells), antigen-presenting cells (7.3% of total cells), macrophages (3.3% of total cells), enterocytes (7.7% of total cells), FABP1^+^ enterocytes (16.2% of total cells), and goblet cells (5.2% of total cells) (**Figure 3B**). These cell populations were consistently identified across both treatment groups and individual replicates (**Figures 3C, D**). Based on lineage identity, these cells were grouped into three categories: lymphocytes(Clusters 0, 2, 4, and 7), myeloid cells (antigen-presenting cells and macrophages), and epithelialcells (enterocytes, FABP1^+^ enterocytes, and goblet cells) (**Supplementary Figure 4**). Myeloid and epithelial cells can be co-isolated with IELs during the isolation process; therefore, we retained these fractions to study their dynamics in response to *S.* Enteritidis infection. The lymphocyte clusters were extracted from the broader dataset and re-clustered to further phenotype T cells.

#### 3.4.1 Myeloid cells

Among the myeloid lineage, antigen-presenting cells were identified by the expression of classical antigen-presenting genes (LYZ, MPEG1, CD74) and innate immune signaling genes (IL23A, and IL18). Macrophages were distinguished due to expression of canonical macrophage markers (MARCO, C1QA, C1QB, C1QC) along with antigen presenting genes (CD74 and CIITA).

#### 3.4.2 Epithelial cells

Two of the clusters were classified as Enterocyte due to expression of canonical enterocyte markers (EPCAM, CDH1, TJP2). One of them also expressed additional genes indicative of maturity (FABP1, FABP2, FABP6, CD36) and thus was designated FABP1^+^Enterocyte. Goblet cells were annotated based on robust expression of classical goblet cell markers (MUC2, SPDEF, AGR2).

#### 3.4.3 Lymphocytes

The four lymphocyte clusters were identified using the canonical markers CD247, CD3D, CD3E, CD8A, CD8BP, LEF1, and TCF7. These clusters were grouped into a single cluster to elucidate the overall trend in the IEL response.

### 3.5 Sub-clustering of the lymphocyte group identified eight IEL subpopulations

**Figure 4:**
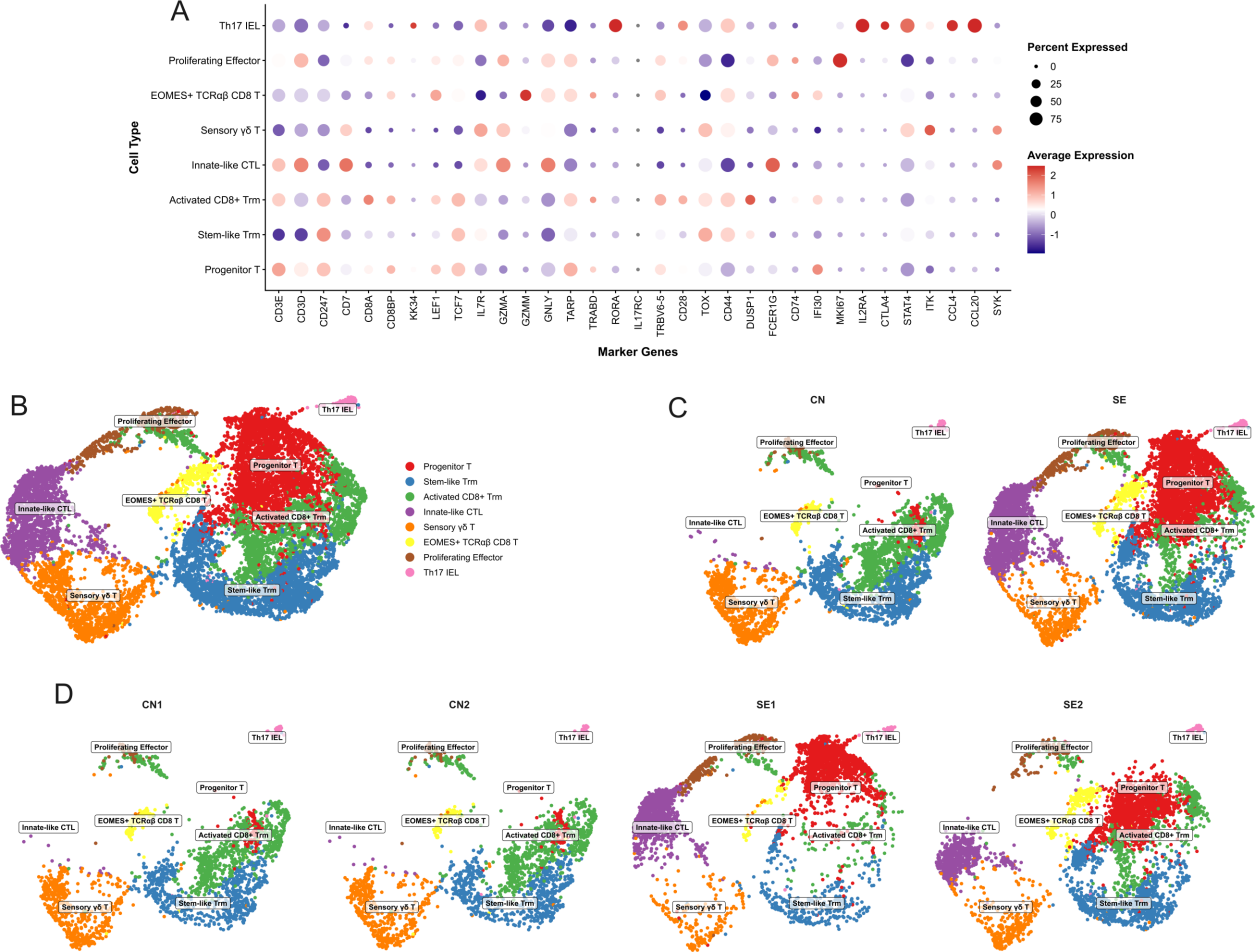
Sub-clustering of the lymphocyte population/IELs from ileal intraepithelial cells of control and *S.* Enteritidis-challenged chickens at 2 days post infection (dpi). Twenty-one-day-old SPF layer chickens were orally gavaged with 1.62 × 10^8^ CFU/bird of *S.* Enteritidis (challenge group, SE) or PBS (control group, CN). The lymphocyte cluster identified in the global clustering analysis (**Figure 3**) was extracted and re-clustered using the Seurat pipeline. **(A)** Dot plot showing marker gene expression across lymphocyte subpopulations. Dot size represents the percentage of cells expressing each gene, and color intensity indicates the average expression level. **(B)** UMAP visualization of all identified lymphocyte subpopulations across both groups: progenitor T, stem-like Trm, activated CD8^+^ Trm, innate-like CTL, sensory γδ T, EOMES^+^ TCRαβ CD8 T, proliferating effector, and Th17 IEL. **(C)** UMAP plots split by treatment group (CN and SE). **(D)** UMAP plots split by individual replicates (CN1, CN2, SE1, and SE2).

To characterize T cell subtypes, the lymphocyte population was extracted and re-clustered using the top 50 differentially expressed genes (DEGs) between clusters, along with the expression of canonical marker genes (**Figure 4A**; **Supplementary Data Sheet 2**). Reclustering of the lymphocyte fraction after removal of contaminating erythrocytes and antigen-presenting cells identified eight lymphoid-lineage clusters (**Figure 4B**). Reclustering was performed using the first 15 principal components and resolution 0.2,based on the elbow plot, and the optimal number of clusters was determined by the Silhouette width (**Supplementary Figure 5**). Resolution was optimized with the help of clustree v0.5.1 (**Supplementary Figure 6**).

#### 3.5.1 Progenitor T cell

Progenitor T cell (Progenitor T) was classified based on the detection of LEF1 and TCF7, whichare involved in T cell development, maturation, and memory, along with the expression of SOX4, which maintains an undifferentiated or progenitor state, and ZNF593, a progenitor-associated transcription factor (**Supplementary Figure 7B**). ZAP70 and CSK genes were also expressed, indicating TCR signaling and further confirming that the cells are of the T cell lineage. Furthermore, the cluster showed increased protein synthesis (RPS26, RPS24, RPL34, RPL31), indicative of an actively translating effector poised state.

#### 3.5.2 Activated effector-resident memory CD8^+^ TCRαβ T cells (Activated CD8^+^ Trm)

Activated effector-resident memory CD8^+^ TCRαβ T cells (ActivatedCD8^+^ Trm) had expression of TRBV6–5 and CD8A, indicating CD8^+^ TCRαβ lineage, along with TCR signaling markers implicating activation state (CD83, EGR3, CRTAM, TNIP2, and HSPB9; **Supplementary Figure 7C**).

#### 3.5.3 Stem-like resident memory T cells (Stem-like Trm)

Stem-like resident memory T cells (Stem-like Trm) were identified based on expression of T cell-associated genes (BCL11B, NFATC1, CD247), genes associated with memory (TOX, CD44), along with genes indicating stem-like nature: TCF7, FOXP1, and FOXO1 (**Supplementary Figure 7D**). Notably, TCF7 is shared between progenitor T and Stem-like Trm, reflecting their sharedstemness characteristic. However, this cluster is distinguished from progenitor T based on enrichment of FOXP1 (significantly lower in progenitor T; Wilcoxon test [adjusted *P* < 0.05]) and FOXO1 (absent in progenitor T; Wilcoxon test [adjusted *P* < 0.05]), two transcription factors that enforce T cell quiescence and long-term self-renewal. It is important to note that progenitor T cells and Stem-like Trm share the TCF7 marker, and individual gene feature plots show overlapping signals between these clusters (**Supplementary Figure 7**), reflecting their transcriptomic similarity, which is further explored through trajectoryanalyses in the following sections. To confirm that the overlap between Stem-like Trm and ProgenitorT was not due to dissociation, we performed a dissociation-induced stress module score using the canonical signature of immediate early genes, heat shock proteins, and stress-response factors (28) across stem-like Trm and progenitor T clusters using Seurat’s AddModuleScore function (**Supplementary Figure 8**). Stem-like Trm and Progenitor T cells exhibited dissociation stress scores tightly distributed around zero (median scores ≈0.05 and ≈0.13, respectively). This confirmed that dissociation-induced stress was not responsible for the transcriptomic similarity between these two clusters.

#### 3.5.4 Innate-like cytotoxic T cells

Expressions of cytotoxic genes (GZMA, GZMK, GNLY) along with T cell specific genes (CD7, and CD3D) denoted cytotoxic T cells. Moreover, expression of NK and innate-linked genes (FCER1G, SYK) was also observed; therefore, we termed this cluster innate-like cytotoxic T cells (Innate-like CTL).

#### 3.5.5 Sensory γδ T cells Sensory gd T

Sensory γδ T cells (Sensory γδ T) were identified by co-expression of SYK and ITK (γδ cells’ hallmark) along with upregulation of HTR4 and ADRB2, which signify neuro-immune crosstalk.

#### 3.5.6 EOMES^+^TCRαβ CD8 T cells

EOMES, GZMM, TRBV6-5, ADORA2A, and TEC expression enabled us to identify this cluster as EOMES^+^TCRαβ CD8 T cells (EOMES^+^TCRαβ CD8 T). EOMES is a defining transcription factor for CD8^+^ effector and memory differentiation, while ADORA2A regulates T cell function, and TEC maintains low-level survival signaling.

#### 3.5.7 Proliferating effector lymphocytes

Proliferating effector lymphocytes (Proliferating Effector) exhibited strong expression of genes related to proliferation (MKI67, TOP2A, CDC20) and lymphocyte lineage (CD3D, BATF, ARHGDIB).

#### 3.5.8 Th17 IEL

The last cluster was designated Th17 IEL based on strong expression of type 17-associated genes (IL17A, IL22, and IL23R) and RORA, which drives the Th17 program in chickens. This cluster also expressed T-cell markers (CD28, ICOS, CD40LG). Furthermore, expression of ERNI (ENSGALG00010028006) and ZBTB46 within this cluster suggested an innate-like characteristic.

### 3.6 Altered DEGs and composition in global clustering following *S.* Enteritidis

**Figure 5:**
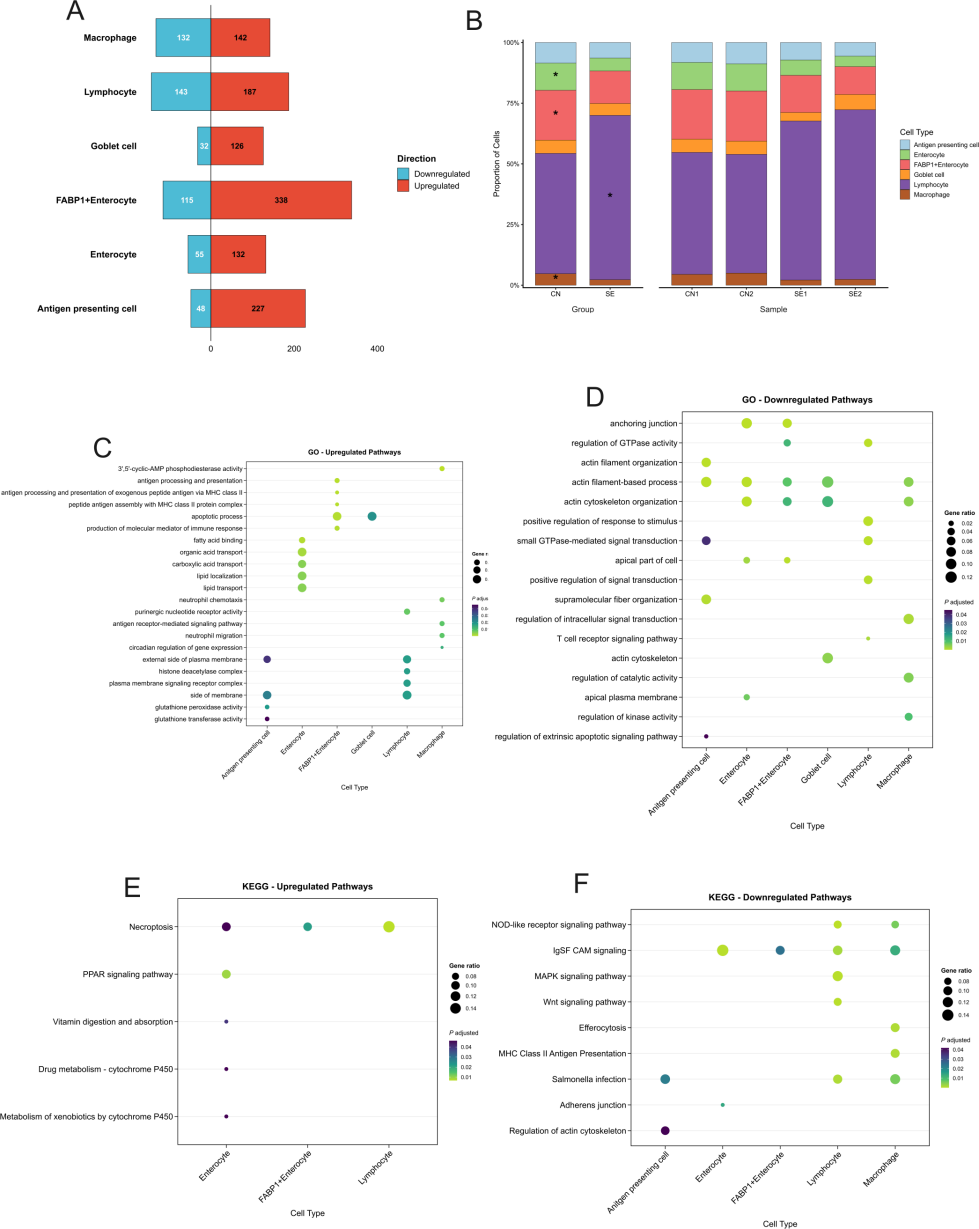
Differential gene expression, cell proportion, and pathway enrichment analyses of global ileal intraepithelial cell types following *S.* Enteritidis challenge at 2 days post infection (dpi). Twenty-one-day-old SPF layer chickens were orally gavaged with 1.62 × 10^8^ CFU/bird of S. Enteritidis (challenge group, SE) or PBS (control group, CN). DEGs between SE and CN were identified within each cell type from the global clustering analysis (**Figure 3**) using the MAST test with CPM normalization and CDR as a covariate (adjusted *P* < 0.05; |log_2_FC| > 0.5). **(A)** Number of upregulated and downregulated DEGs per cell type. **(B)** Proportion of cells contributed by each cell type in CN and SE groups (left) and across individual replicates (CN1, CN2, SE1, and SE2; right). Cell proportion differences were assessed using propeller (* indicates adjusted *P* < 0.05). **(C)** GO enrichment analysis of upregulated DEGs across cell types. **(D)** GO enrichment analysis of downregulated DEGs across cell types. **(E)** KEGG pathway enrichment analysis of upregulated DEGs across cell types. **(F)** KEGG pathway enrichment analysis of downregulated DEGs across cell types. In panels C–F, dot size represents the gene ratio and color intensity indicates the adjusted *P*-value.

To study transcriptomic differences, DEGs were compared between the CN and SE groups for each cell type. The largest number of upregulated genes was observed in FABP1^+^ enterocytes, followed by lymphocytes and antigen-presenting cells (**Figure 5A**). The cellular composition of ileal epithelial compartments differed between control (CN) and *S.* Enteritidis-challenged (SE) groups (**Figure 5B**). Lymphocytes represented the dominant population and were significantly increased in the SE group compared to CN (*P* adj < 0.05). In contrast, enterocytes, FABP1^+^ enterocytes, and macrophages were significantly reduced in SE relative to CN (*P* adj < 0.05). These patterns were consistent across biological replicates (CN1, CN2, SE1, SE2), indicating reproducibility of the observed changes (**Figure 5B**).

### 3.7 *S.* Enteritidis challenge induced a significant shift in translation and metabolic activity

**Figure 6:**
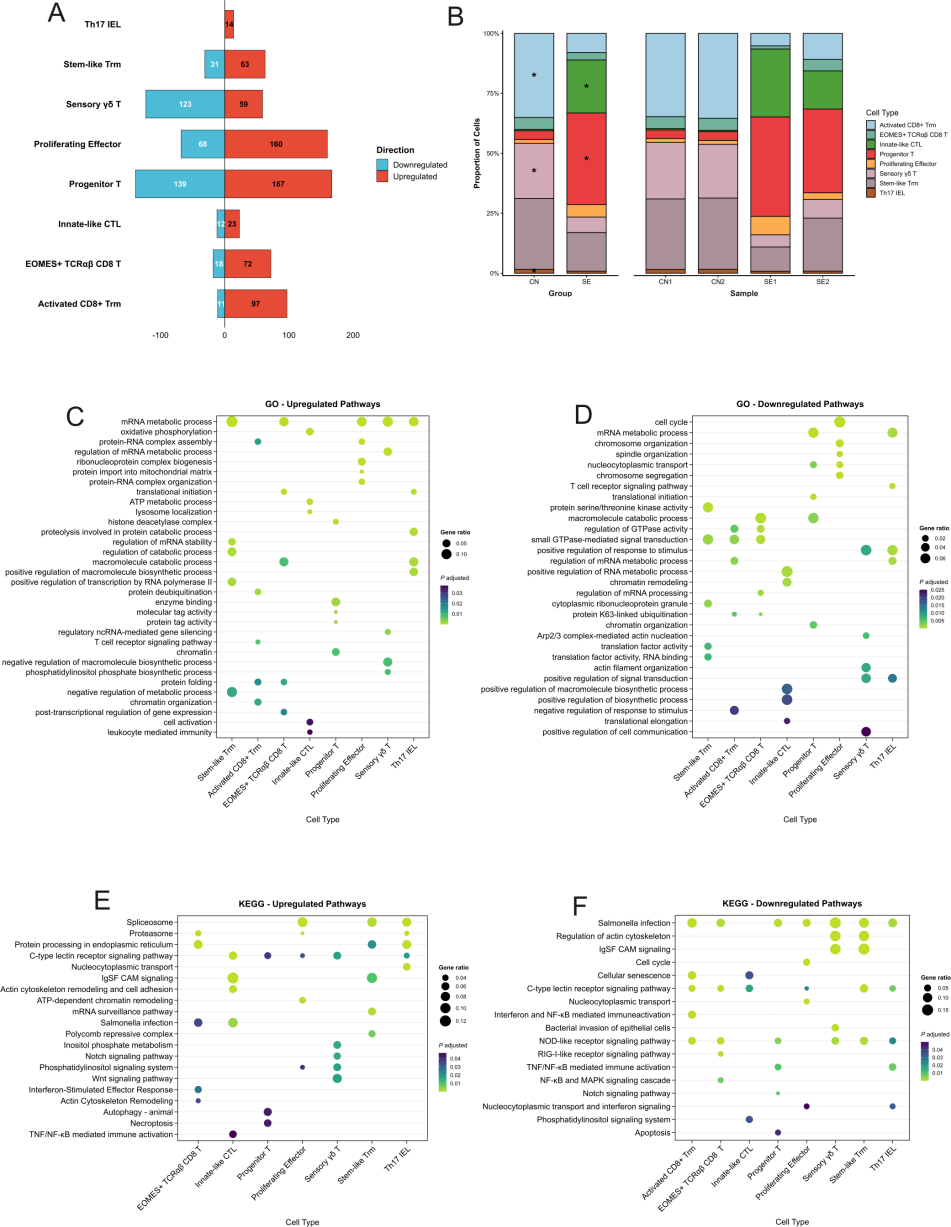
Differential gene expression, cell proportion, and pathway enrichment analyses of ileal IELs/lymphocyte subpopulations following *S.* Enteritidis challenge at 2 days post infection (dpi). Twenty-one-day-old SPF layer chickens were orally gavaged with 1.62 × 10^8^ CFU/bird of S. Enteritidis (challenge group, SE) or PBS (control group, CN). Differentially expressed genes (DEGs) between SE and CN were identified within each lymphocyte subpopulation (**Figure 4**) using the MAST test with counts per million (CPM) normalization and cellular detection rate (CDR) as a covariate (adjusted *P* < 0.05; |log_2_FC| > 0.5). **(A)** Number of upregulated and downregulated DEGs per lymphocyte subpopulation. **(B)** Proportion of cells contributed by each lymphocyte subpopulation in CN and SE groups (left) and across individual replicates (CN1, CN2, SE1, and SE2; right). Cell proportion differences were assessed using propeller, * indicates adjusted *P* < 0.05. **(C)** Gene Ontology (GO) enrichment analysis of upregulated DEGs across lymphocyte subpopulations. **(D)** GO enrichment analysis of downregulated DEGs across lymphocyte subpopulations. **(E)** KEGG pathway enrichment analysis of upregulated DEGs across lymphocyte subpopulations. **(F)** KEGG pathway enrichment analysis of downregulated DEGs across lymphocyte subpopulations. In panels **(C–F)**, dot size represents the gene ratio, and color intensity indicates the adjusted *P*-value.

Across both clustering levels, cells in the SE group showed coordinated upregulation of ribosomalprotein (e.g., RPL and RPS) and metabolic pathway genes, indicating elevated translational capacityand increased energy production to support immune system modulation and cellular turnover. Similar findings have been reported in chicken lymphocytes following *S.* Enteritidis challenge by Sekelova et al. (29) where they reported an increase in proteins related to the ribosome and oxidative phosphorylation. Furthermore, *Salmonella* challenge studies in murine model also reported metabolic reprogramming and upregulation of certain metabolic pathways and ribosomal biogenesis (30, 31). Based on these DEGs findings and focusing on immune-specific pathways, we filtered out the common genes related to translation and metabolism. Subsequent GO and KEGG analyses prioritized immune-related and infection-associated pathways (*P* adj < 0.05). Full details for GO and KEGG pathways are provided in **Supplementary Data Sheet 3** (**Figures 5C–F**) and **Supplementary Data Sheet 4** (**Figures 6C–F**).

Looking at initial clustering, FABP1^+^ enterocytes showed coordinated upregulation of mitochondrial respiratory chain components (NDUF, UQCR, COX, and ATP5 families), indicating enhanced oxidative phosphorylation. Pathway enrichment revealed upregulation of antigen presentation and necroptosis, alongside downregulation of IgSF CAM signaling (**Figures 5C–F**), which is involved in the heterophils’ recruitment (32), and intestinal barrier maintenance through CADM1 (33). Macrophages exhibited enhanced oxidative phosphorylation and immune activation signaling (MIF, MAP1LC3B, FCER1G) but also showed downregulation of the KEGG “*Salmonella* infection” pathway and MHC Class II antigen presentation (**Figures 5C–F**). Genes in the hampered *Salmonella* infection pathway correlated with the chaperone pathway, which interrupts *Salmonella* vacuole maturation and assists phagocytosis. These findings indicate that *Salmonella* depresses specific pathways to evade the immune system, which is consistent with well documented *Salmonella* strategies to impair macrophage function and promote intracellular persistence (34, 35).

### 3.8 *S.* Enteritidis challenge altered gene expression and drove substantial expansion of progenitor T cells and innate-like CTL

In lymphocyte sub-clustering, progenitor T cells had the highest number of DEGs, followed by the proliferating effector cell type (**Figure 6A**). Comparison of IEL composition between CN and SE birds revealed striking differences in lymphocyte subset distribution (**Figure 6B**). Progenitor T, which comprised 3.6% of lymphocytes in CN birds, expanded to 38% following *Salmonella* challenge (*P* adj < 0.05). In parallel, innate-like CTLs, which were nearly absent in CN birds (0.5%), emerged as a substantial population in the SE group (21.5%; *P* adj < 0.05; **Figure 6B**). Among individual replicates, a similar pattern was observed, indicating reproducibility (**Figure 6B**). In light of the flow cytometry findings, this shift is noteworthy. Whereas flow cytometry showed an increase in the proportion of the TCRγδ^+^ CD8αβ^+^ phenotype, scRNA-seq displayed a more nuanced response, highlighting plasticity in the IEL compartment.

In contrast, activated CD8^+^ Trm, Sensory γδ T, and Th17 IEL were present in higher proportions in the CN group (*P* adj < 0.05), whereas the remaining subsets were relatively comparable between the groups.

### 3.9 Progenitor T cells shifted toward a cytotoxic effector program, while innate-like CTL displayed TCR-independent effector signatures

Given the substantial expansion of progenitor T cells and innate-like CTL in the SE group, we focused on their immune-related transcriptomic profiles.

Progenitor T cells in the SE group demonstrated a transition toward a cytotoxic phenotype, as evidenced by upregulation of XCL1, GNLY, and CD7. Furthermore, downregulation of GFI1, ID3, and RUNX1 confirmed suppression of progenitor state. Pathway enrichment indicated activation of innate inflammatory program (C-type lectin receptor signaling, autophagy, necroptosis) (**Figures 6C–F**). Moreover, KEGG enrichment revealed downregulation of gene sets annotated under ‘*Salmonella* infection’, driven primarily by decreased expression of cytoskeletal/adhesion and MAPK-related genes (e.g., ACTB/ACTG1, RHOA/CDC42, RAP1A/B, MAPK3), suggesting infection-induced remodeling of motility/adhesion and signaling structure in progenitor IELs.

Innate-like CTL were extremely rare in control birds (13 cells total) but abundant in infected birds (900 cells), preventing direct within-cluster DEGs analysis between groups. Instead, we characterized this population’s functional identity by its cluster defining marker genes relative to other lymphocyte subsets. Innate-like cytotoxic T cells displayed robust expression of granule-mediated effector machinery, including GZMA, GZMK, PRF1, GNLY, FASLG, and CST7, consistent with active cytolytic competence. These cells exhibited a signaling architecture enriched for innate adaptors (FCER1G, SYK, SH2D1B, LYN) and downstream PI3K–MAPK components, indicating a shift toward innate receptor–mediated activation. Pathway analysis indicated upregulation of TNF/NF-κB-mediated immune activation, the C-type lectin receptor signaling pathway, and genes related to *Salmonella* infection (**Figures 6C–F**). The overall signature indicated cytotoxicity and rapid TCR-independent response to *Salmonella* in the intestinal epithelium.

### 3.10 Trajectory analysis inferred a robust transcriptional trajectory toward innate-like CTL

**Figure 7:**
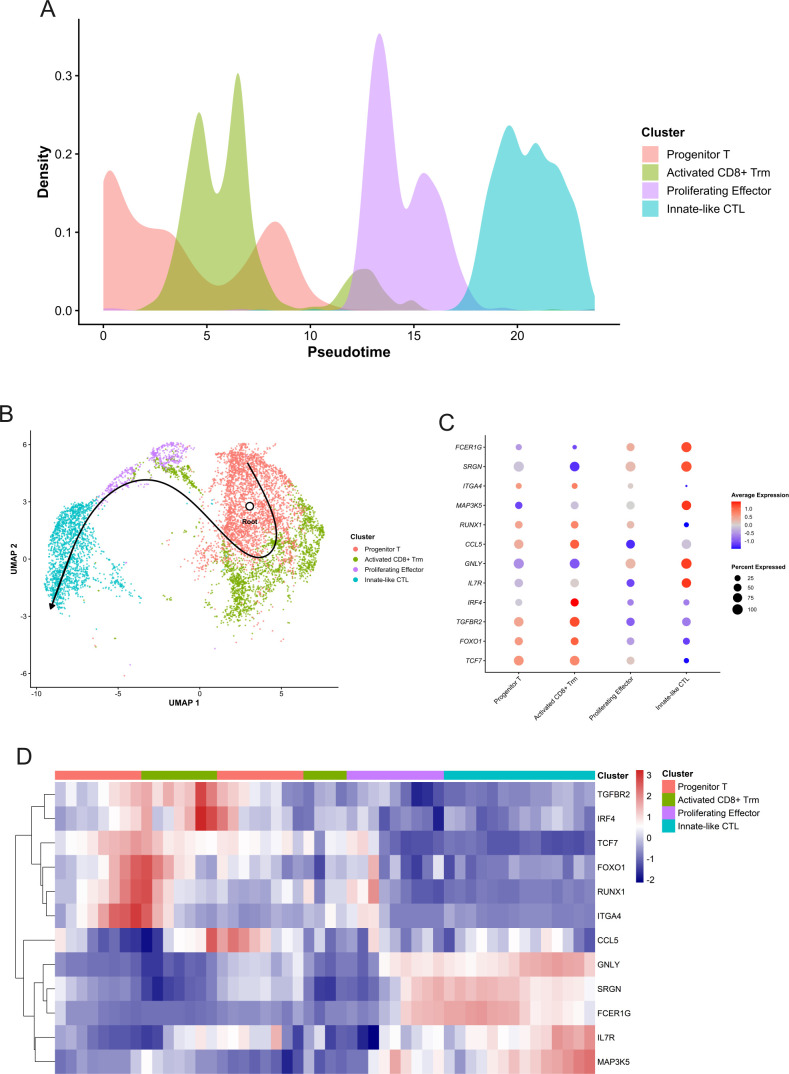
Trajectory analysis of Lineage 1 from ileal IELs/lymphocyte subpopulations at 2 days post infection (dpi). Twenty-one-day-old SPF layer chickens were orally gavaged with 1.62 × 10^8^ CFU/bird of *S.* Enteritidis (challenge group, SE) or PBS (control group, CN). Trajectory analysis was performed on lymphocyte subpopulations identified in **Figure 4** using Slingshot. Lineage 1 represents an inferred transcriptional trajectory ordered from progenitor T cells, through activated CD8^+^ Trm and proliferating effector cells, to innate-like CTL. The root was assigned to the progenitor T cluster based on biological reasoning. **(A)** Density plot showing the distribution of each cluster along pseudotime. **(B)** UMAP visualization of Lineage 1 with the inferred trajectory overlaid. **(C)** Dot plot showing the expression of selected genes across the four clusters comprising Lineage 1. Dot size represents the percentage of cells expressing each gene, and color intensity indicates the average expression level. **(D)** Heatmap of gene expression ordered along pseudotime, with cells colored by cluster identity.

To infer the transcriptional ordering of clusters relative to innate-like CTL, we performedtrajectory analysis using Slingshot on lymphocyte subpopulations, excluding Th17 IEL and sensory γδ T cells due to their distinct biological lineages. The analysis identified three lineages, with the Progenitor T cluster as the origin (**Supplementary Figure 9A**). Lineage 1, which started at Progenitor T and progressed through Activated CD8^+^ Trm and proliferating effector before terminating at Innate-like CTL (**Figures 7A–D**) produced the most robust trajectory, comprising 7,877 cells with minimal off-trajectorycontamination (**Supplementary Figure 9B**). Lineage 2 and lineage 3 terminating at Stem-like Trm and EOMES^+^TCRαβ CD8 T, respectively, showed substantial off-trajectory contamination and were not pursued further (**Supplementary Figure 9B**). Notably, across all lineages, activated CD8^+^ Trm cells represented a common branch point at which gene expression diverged, toward distinct cluster identities along the inferred trajectories.

Pseudotime density analysis of Lineage 1 confirmed the sequential ordering of clusters along the trajectory (**Figure 7A**), and a heatmap of gene expression along pseudotime revealed a gradient of cytotoxic and innate-like transcriptional programs across the inferred trajectory (**Figure 7D**). Selected marker genes further supported the transcriptional gradient between progenitor and effector identities (**Figure 7C**).

**Figure 8:**
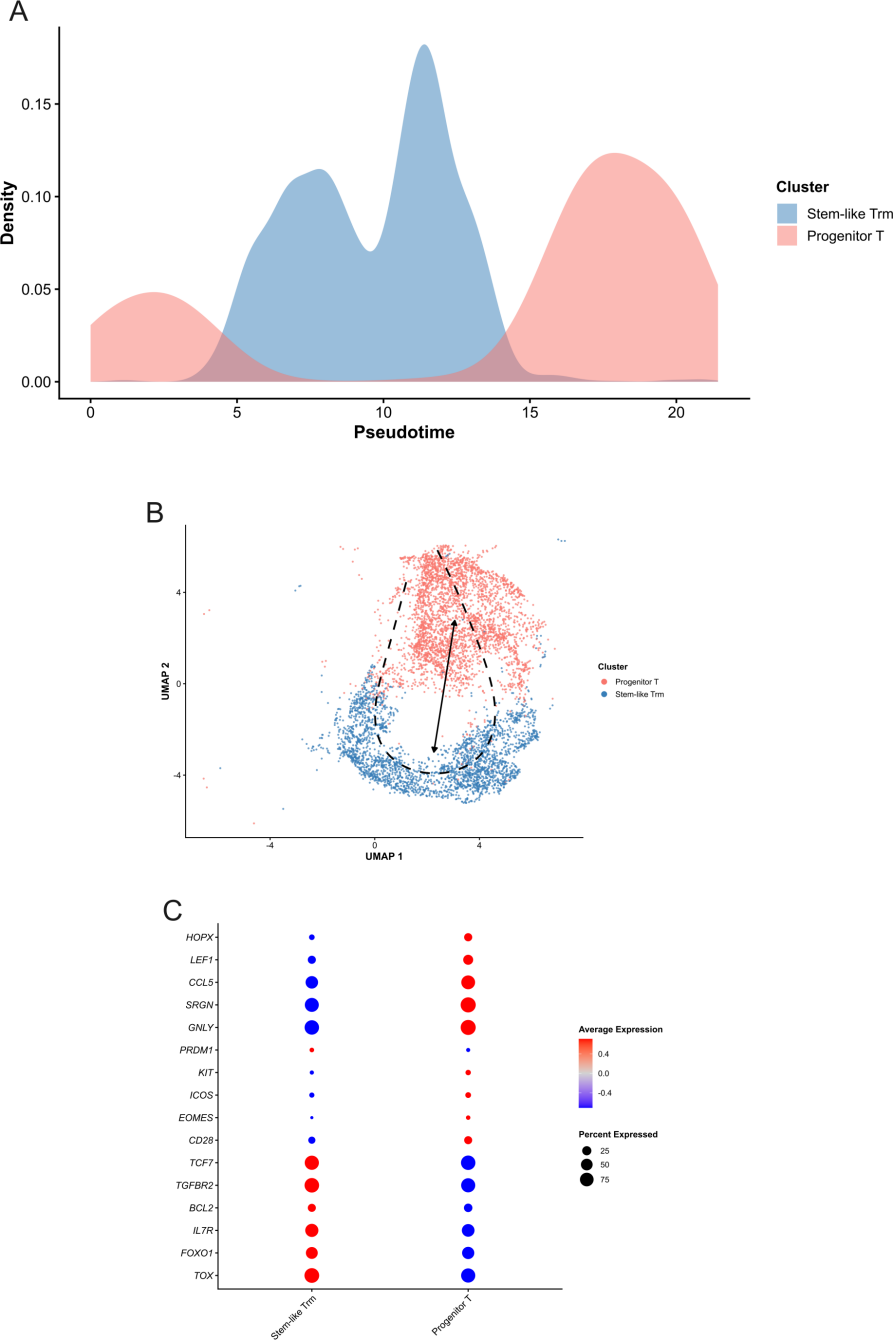
Trajectory analysis of stem-like Trm and progenitor T clusters from ileal IEL subpopulations/lymphocytes at 2 days post infection (dpi). Twenty-one-day-old SPF layer chickens were orally gavaged with 1.62 × 10^8^ CFU/bird of *S.* Enteritidis (challenge group, SE) or PBS (control group, CN). To investigate the potential transition between stem-like Trm and progenitor T cells, a focused trajectory analysis was performed on these two clusters (identified in **Figure 4**) using Slingshot. **(A)** Density plot showing the distribution of each cluster along pseudotime, revealing extensive overlap with no clear unidirectional gradient, indicative of transcriptomic plasticity between quiescence and an effector-ready state. **(B)** UMAP visualization of the inferred trajectory between stem-like Trm and progenitor T clusters. **(C)** Dot plot showing the expression of selected genes across the two clusters. Dot size represents the percentage of cells expressing each gene, and color intensity indicates the average expression level.

To investigate the relationship between quiescent stem-like and progenitor T cells during infection, a focused trajectory analysis was performed on these two clusters, given their transcriptomic similarities. Pseudotime density analysis revealed extensive overlap between the populations, with no clear unidirectional gradient (**Figures 8A–C**). This revealed that the two clusters overlapped, depicting transcriptomic plasticitybetween quiescence and an effector-ready state. Gene sets used for trajectory inference are listedin **Supplementary Data Sheet 5**.

## 4 Discussion

Intraepithelial lymphocytes occupy a strategic position within the intestinal epithelium, forming the first line of defense against enteric pathogens. Despite their importance, the cellular heterogeneity and functional response of chicken IELs to *Salmonella* infection have remained poorly characterized at the single-cell level. To our knowledge, this study performed the first single-cell transcriptomic analysis of chicken IELs responding to *Salmonella* Enteritidis infection, complemented by spectral flow cytometry to gain deeper insight into IEL subset dynamics and transcriptional program at cellular resolution.

Our challenge model established a subclinical infection, as evidenced by cecal colonization and systemic spread to the liver without clinical signs or mortality, indicating a carrier state frequently reported with *Salmonella* infection in chickens (25–27). From a foodborne pathogen perspective, this subclinical infection is a major drawback, due to difficulty in diagnosis and risk of food chain contamination driven by *Salmonella*’s ability to evade and suppress the immune response (36, 37).

Both flow cytometry and scRNA-seq converged on the same central finding that *S.* Enteritidis challenge drives an early cytotoxic IEL response. Flow cytometry demonstrated a transient increase in the overall IEL population, specifically TCRγδ^+^ CD8αβ^+^ IELs. Concurrently, scRNA-seq revealed a substantial expansion of innate-like cytotoxic T cells and progenitor T cells, which displayed a cytotoxic signature following the challenge. These complementary findings are supported by prior literature reporting an increase in CD8^+^ TCRγδ^+^ IELs and enhanced cytotoxicity during murine *Salmonella* Typhimurium challenge (38). These findings are further supported by increased intestinal infiltration by IELs at 1 dpi with *S.* Enteritidis in chickens (39) and partially supported by our previous study, which reported increases in multiple IEL subsets, including the cytotoxic subset, at 2 and 7 dpi following broiler chicken *Salmonella* Typhimurium infection (15). The limited number of IEL subsets observed in the current study, compared to our previous work, may be due to differences in *Salmonella* serovar and bird strain.

Our finding of expanded cytotoxic TCRγδ^+^CD8αβ^+^ IELs during *S*. Enteritidis challenge mirrors the well characterized response of mammalian intestinal γδ T cells to bacterial pathogens. In mice, intestinal γδ IELs constitute a substantial fraction of the gut epithelial lymphocyte compartment and exhibit constitutive cytotoxic activity through granzyme and perforin expression, often via TCR-independent activation (1) and have been implicated in mucosal resistance to *Salmonella* infection (40). In humans, intestinal γδ T cells subset display innate-like effector phenotypes and rapidly respond to enteric pathogens via NKG2D and butyrophilin-mediated pathways (2, 41). The cytotoxic γδ IEL expansion we observed in chicken following enteric *Salmonella* challenge thus aligns with an evolutionarily conserved role for intestinal γδ T cells in early mucosal defense against bacterial pathogens, despite the substantial differences in γδ T cell repertoire diversity between birds and mammals.

Integrating our scRNA-seq and trajectory analysis findings, we propose the following model for IEL dynamics during *S.* Enteritidis infection in chicken:

Under physiological conditions, thymic-derived stem-like Trm cells, characterized by expression of FOXP1, TCF7, FOXO1, and TOX, constitute the dominant IEL population and are maintained in a quiescent state within the intestinal epithelial environment. This is consistent with mammalian studies demonstrating that the majority of IELs derive from thymic progenitors that migrate to the gut for maturation (42), with thymic output being the dominant source of T-lineage IEL populations (43).

Upon *S.* Enteritidis challenge, the transcriptional relationship between stem-like Trm and progenitor T cells may shift toward the progenitor T state, which exhibit a hybrid or poised state, simultaneously expressing stem cell genes (TCF7, LEF1) and effector associated genes alongside elevated ribosomal activity enabling rapid effector response. A similar hybrid state has been described in human and murine stem memory T cells (44). Trajectory analysis of these two populations revealed overlapping states with no clear unidirectional gradient, consistent with transcriptomic plasticity and with overlapping states between quiescence and an effector-ready identity.

Following activation, the inferred Slingshot trajectory transcriptionally orders progenitor T cells through activated CD8^+^ Trm and proliferating effector stages, with innate-like CTL as the predicted terminal cluster of Lineage 1. This proposed developmental relationship is a working hypothesis supported by pseudotime density analysis showing sequential cluster ordering, and would benefit from future experimental validation through lineage tracing or RNA velocity analysis in chicken IEL systems.

The most striking finding of this study was the emergence of innate-like CTL, which were virtually absent in control birds (13 cells). These cells co-expressed classical T cell markers with granule-mediated effector molecules (GZMA, GZMK, PRF1, GNLY) and innate signaling adaptors (FCER1G, SYK), indicating TCR-independent cytolytic capacity.

In poultry, innate-like cytotoxic IELs have not been investigated extensively but have been described in both murine and human systems. In mice, these cells are classified as TCRαβ^+^ CD8αα^+^, and they express NK cell receptors and Fas-ligand, along with the common TCR (43, 45). However, these cells are not believed to play a role in initiating proinflammatory actions against pathogens and are typically associated with tissue homeostasis and immune regulation (46). In contrast, human innate-like cytotoxic cells are described as TCRαβ^+^ CD8αβ^+^ cells that express NK cell receptors and are capable of TCR-independent responses to epithelial stress ligands (2). Moreover, TCRγδ^+^ CD8αβ^+^ has also been characterized in both humans and mice (47, 48). Sumaria et al. (47) showed these cells produced IFN-γ through STAT5-dependent innate mechanisms rather than TCR mediated activation, and a scRNA-seq study confirmed their expression of cytotoxic features along with NK receptors (49). Our flow cytometry results demonstrated that TCRαβ^+^ CD8αβ^+^ and TCRγδ^+^ CD8αβ^+^ cells constitute a significant portion of the IEL compartment (30% and 24%, respectively [average of both groups and timepoints]; combined, they represent more than 50% of the IEL compartment), and specifically, TCRγδ^+^ CD8αβ^+^ increased in proportion following *Salmonella* challenge. This, combined with the innate-like transcriptional signature identified by scRNA-seq, suggests that chicken innate-like CTL may express a CD8αβ^+^ phenotype rather than a CD8αα^+^ phenotype, and that selective expansion of TCRγδ^+^ CD8αβ^+^ cells may indicate that a proportion of these cells derive from the TCRγδ lineage. Recent mouse studies have also reported that conventional CD8αβ^+^ IELs can be reprogrammed to function as innate-like cytotoxic IELs (50), further supporting the concept of functional plasticity. Since IELs represent the frontline defense, the ability to rapidly mount an innate-mediated cytotoxic response in lieu of waiting for TCR dependent antigen recognition may represent a critical early defense mechanism against enteric pathogens.

Previous studies have reported that NK cells are also a major constituent of the IEL compartment (39, 51, 52). However, a definitive NK cell marker has not been established in chickens, as the 28–4 antibody previously used for NK cell identification has been reclassified as anti-CD25, which is also expressed by T cells and innate lymphoid cells (53). In our scRNA-seq, NK-associated markers were co-expressed with T cell markers within the same clusters, a phenomenon that likely reflects the heightened plasticity of immune cells within the intestinal epithelial environment (54). Consequently, we were unable to definitively resolve whether a distinct NK cell population exists within the chicken IEL compartment using 3′-end scRNA-seq alone.

Several recent scRNA-seq studies have characterized chicken immune cells, primarily focusing on systemic and central lymphoid compartments such as spleen, thymus, and peripheral blood (55–58). The most directly comparable study to ours is by Tu et al. (59), who profiled the chicken cecum and identified intestinal epithelial cells along with the immune cells. They reported multiple T cell types defined by canonical markers shared with our dataset (GZMA, GNLY, CD3E, CD3D, TCF7, LEF1). However, Tu et al. examined whole cecal tissue rather than the intraepithelial compartment specifically, and the cluster annotations they assigned differ from ours. Such differences in cluster nomenclature across studies are expected: scRNA-seq cluster names reflect the cells’ functional state in a particular biological context, and the specific gene programs captured are shaped by the tissue source, infection or experimental condition, and sampling timepoint.

Some limitations should be considered when interpreting these findings. First, scRNA-seq was performed on two pooled samples per group, which limits statistical power for differential abundance and expression analyses. However, the consistency between biological replicates (**Figure 3D**, **4D**), concordance with flow cytometry data (n = 4 per group), and the use of propeller for proportion testing provide confidence in the major findings. Second, scRNA-seq was performed at 2 dpi only, therefore, we could not capture the full temporal dynamics of the IEL response. The flow cytometry data at 6 dpi, which showed resolution of the cytotoxic shift, suggest that the response is transient, and time-course scRNA-seq would be needed to fully characterize these dynamics and evaluate long-term maintenance of immune response. Third, there was variability in the number of cells recovered per scRNA-seq library (5,000–13,000 cells/library). While, such variability is common in droplet-based scRNA-seq from primary tissue, particularly low-yield mucosal compartments, it can affect the sampling depth of rare cell populations and the precision of proportional comparisons between conditions. We mitigated these effects through SCTransform normalization, anchor-based integration of the four libraries into a shared embedding, MAST-based differential expression testing with cellular detection rate as a covariate to control for per-cell technical variation, and propeller for cluster proportion testing, which accounts for library level variance. Cluster identities and marker gene profiles were consistent across libraries (**Figure 3D**, **4D**), supporting the robustness of the major findings.

## 5 Conclusion

In conclusion, this study provides the first single cell transcriptomic characterization of chicken IELs during *S.* Enteritidis infection, revealing innate-like CTL as a crucial early defense mechanism. Through scRNA-seq, we were able to functionally describe various IEL subsets such as stem-like self-renewing Trm cells that can serve as a reservoir for IEL effector differentiation. Future studies should include 5′ scRNA-seq or full-length scRNA-seq to definitively assign TCR identities to the subpopulations described here, and include more time points post-infection. Finally, understanding the signals that drive stem-like Trm activation and innate-like CTL differentiation may inform strategies to modulate IEL responses for enhanced *Salmonella* resistance in poultry, with potential translational implications for mucosal vaccine development and antibiotic free production systems.

## Data Availability

The data presented in the study are deposited in the NCBI GEO repository, accession number GSE326338.
