# Supplementary figures and images for: Single-cell transcriptomic profiling reveals innate-like cytotoxic intraepithelial lymphocyte expansion during Salmonella Enteritidis infection in chickens
Source: Front Immunol. 2026 Jun 3;17:1842283. doi: 10.3389/fimmu.2026.1842283 (PMC13271962; doi:10.3389/fimmu.2026.1842283)

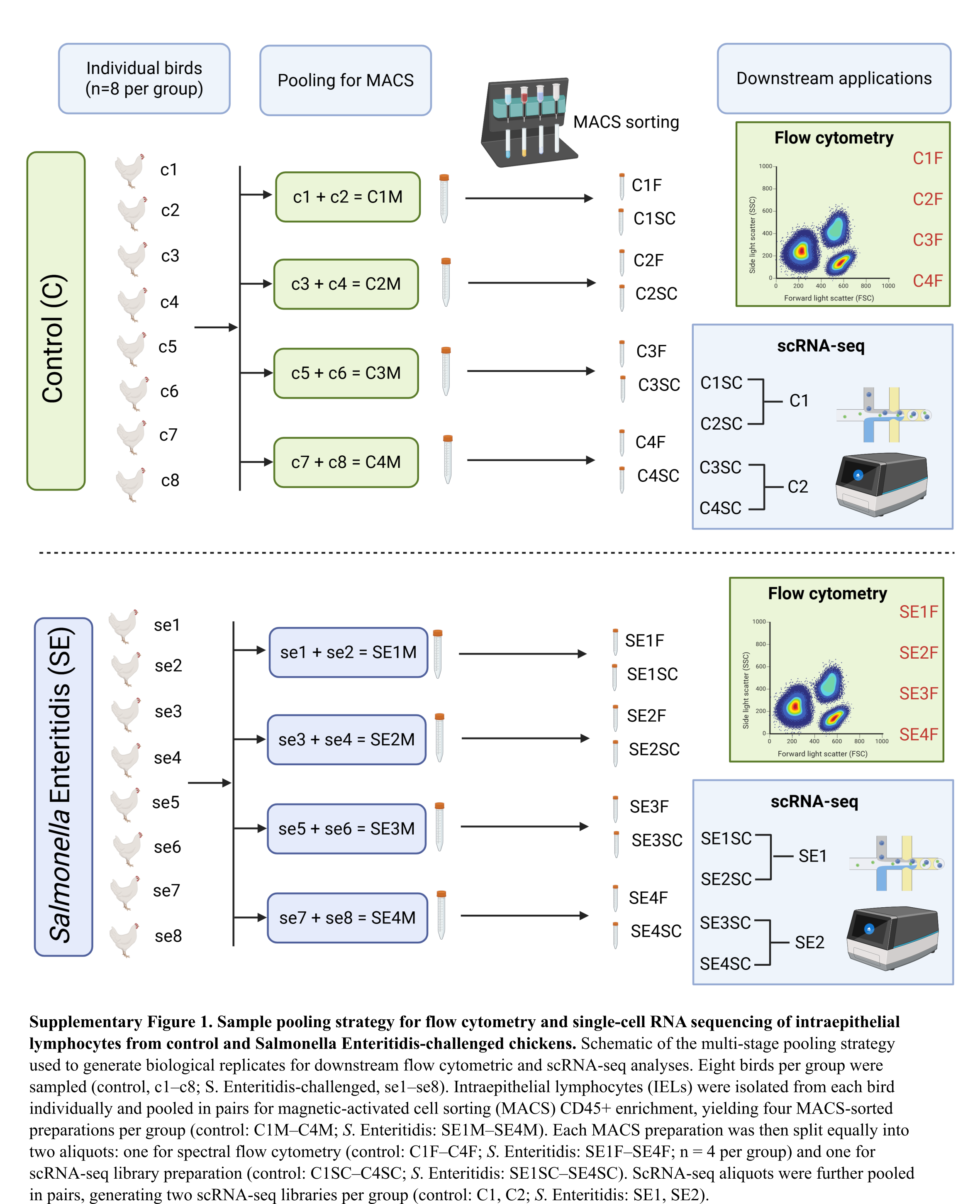

Supplement: Supplementary file 1 [file Image1.tiff]

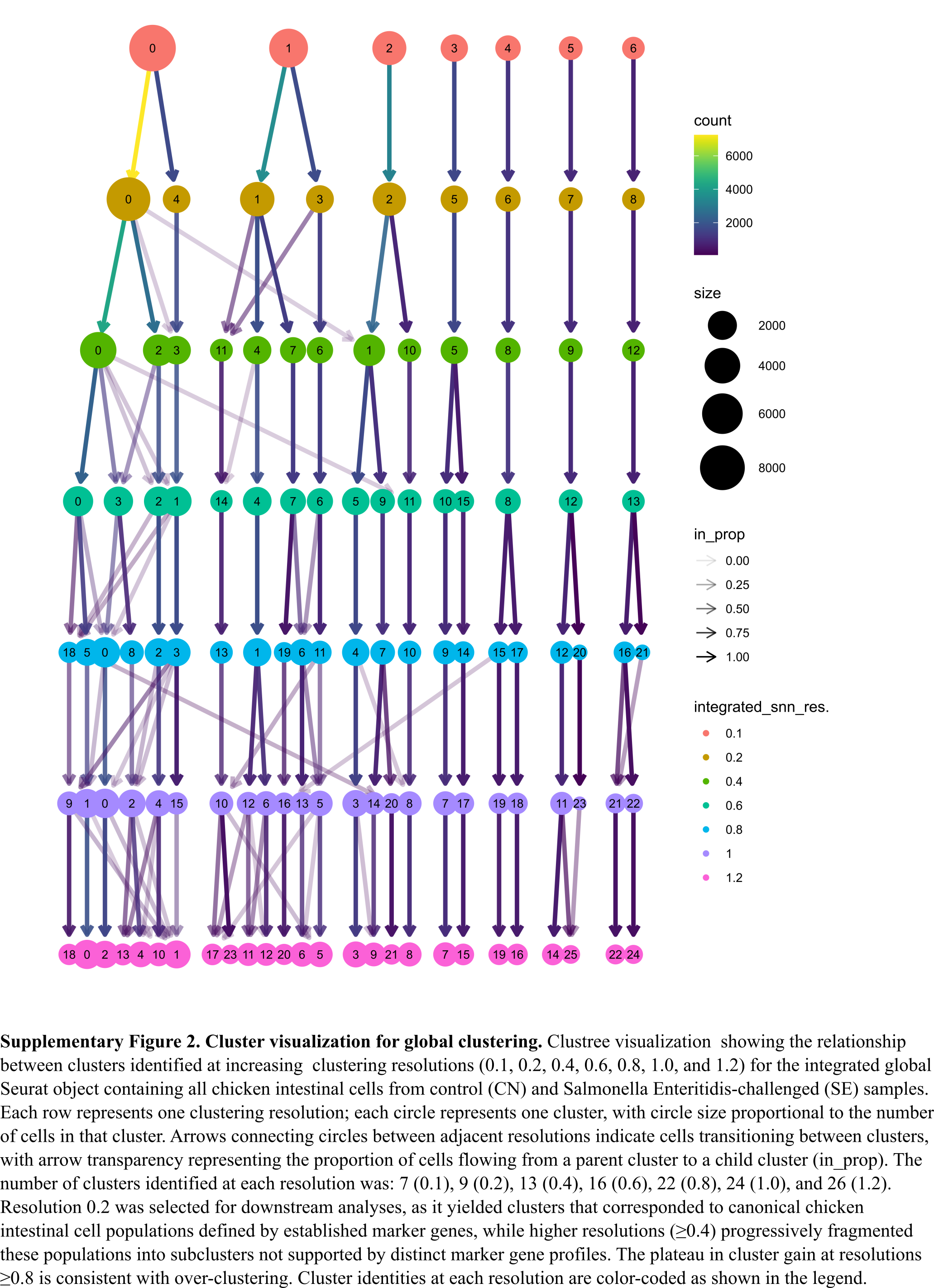

Supplement: Supplementary file 2 [file Image2.tiff]

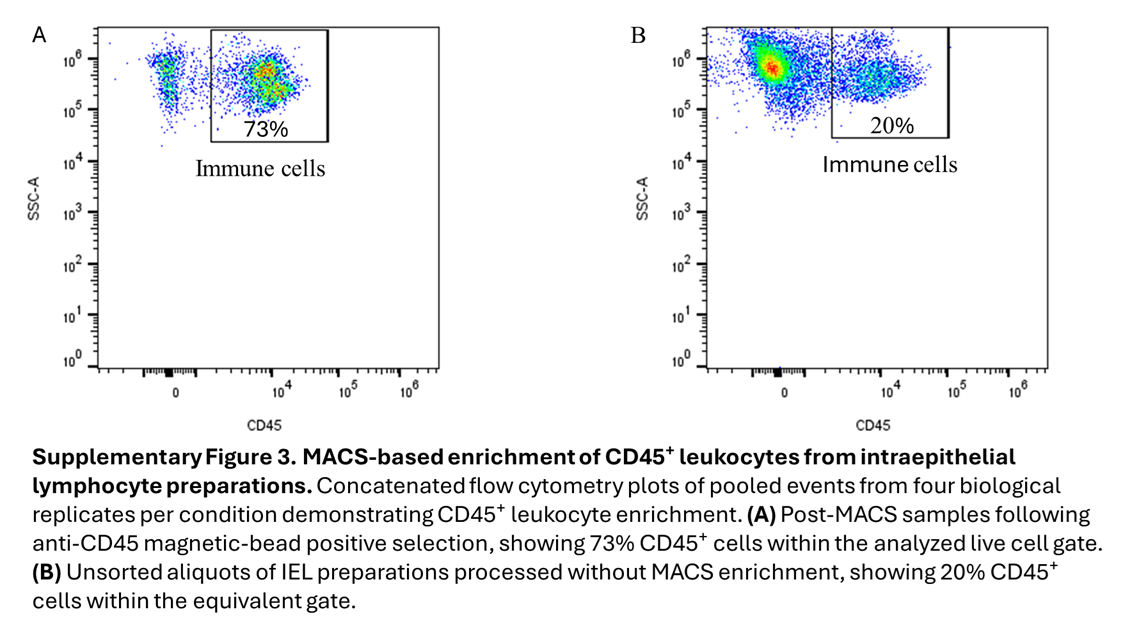

Supplement: Supplementary file 3 [file Image3.tif]

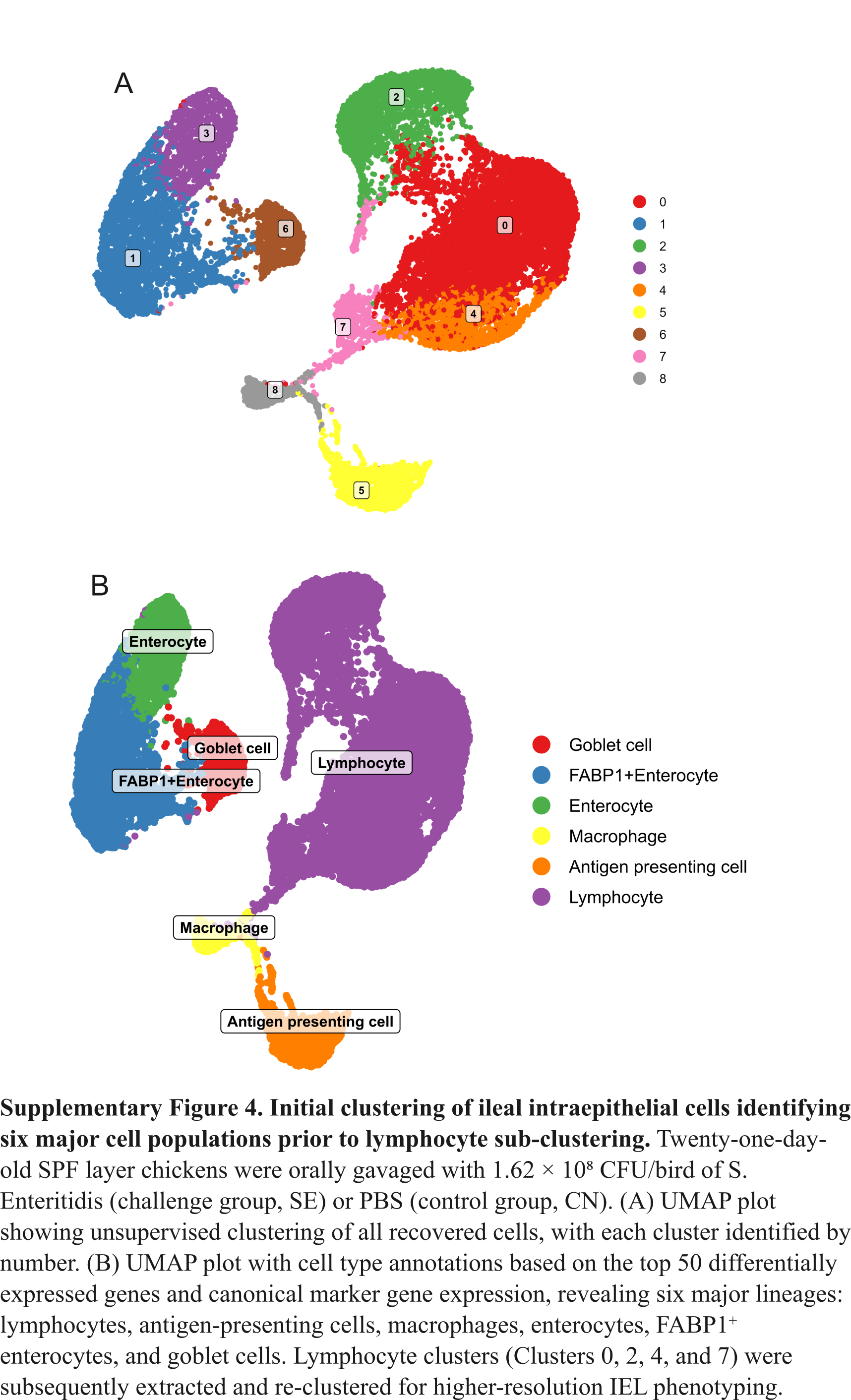

Supplement: Supplementary file 4 [file Image4.tiff]

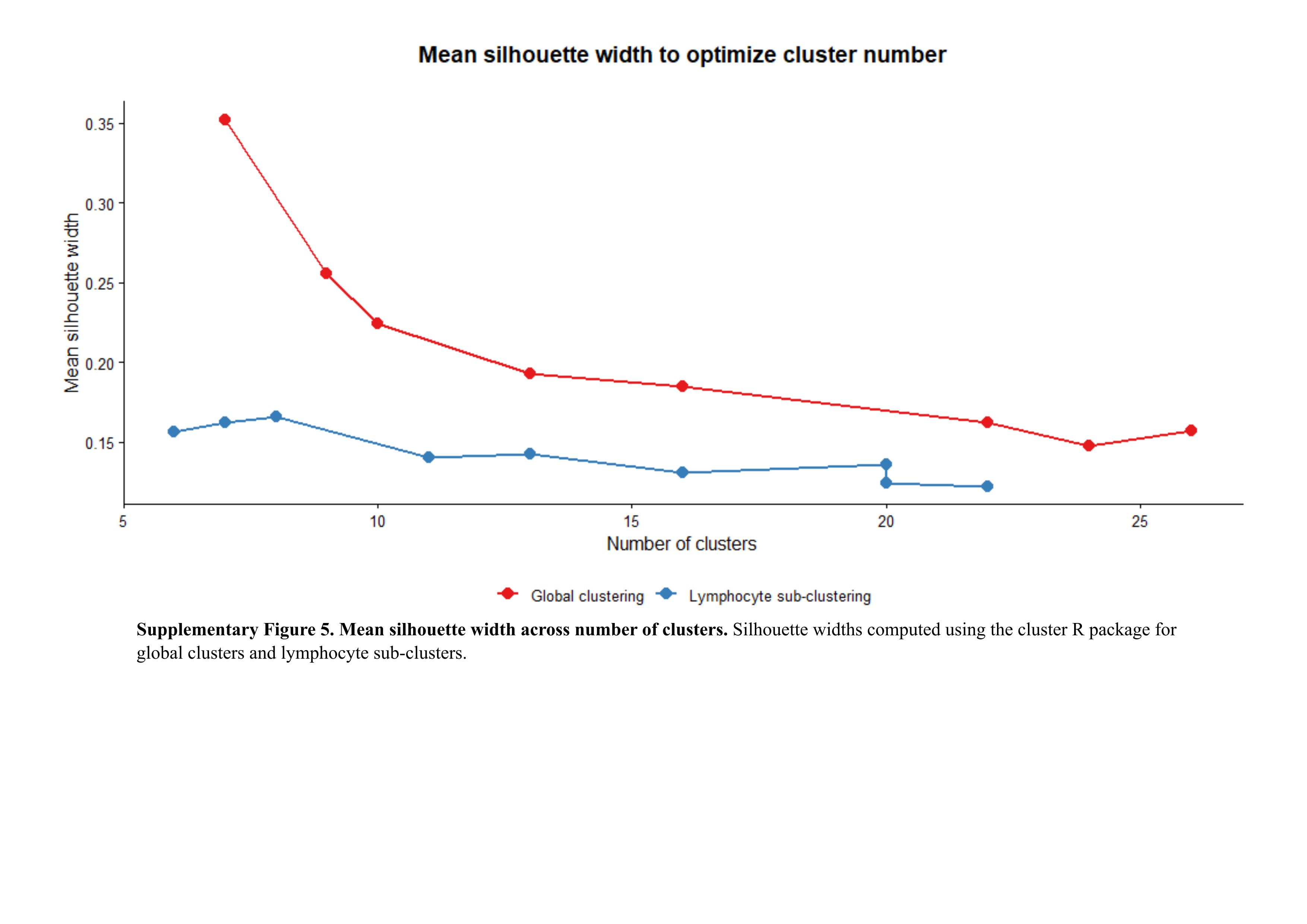

Supplement: Supplementary file 5 [file Image5.tiff]

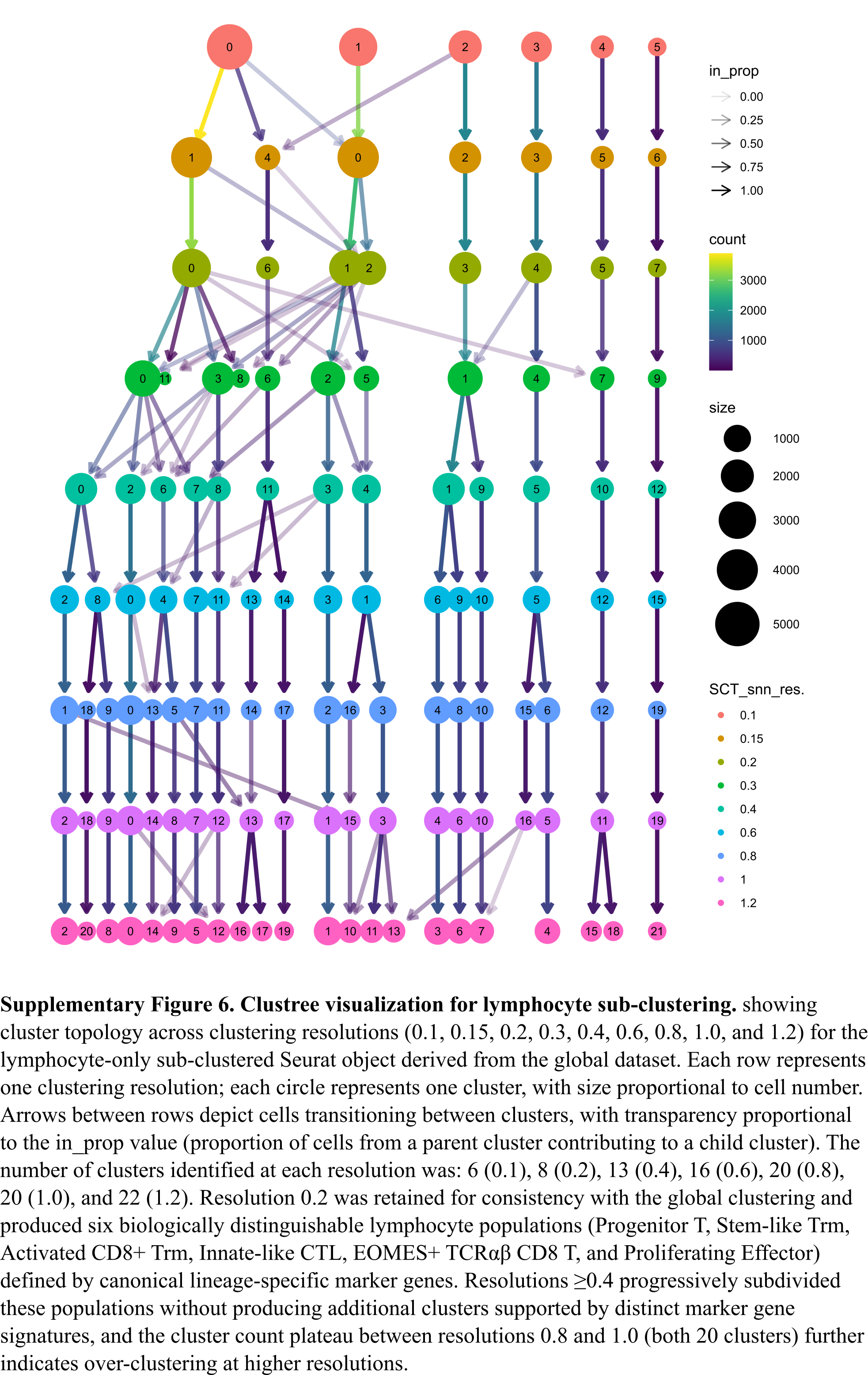

Supplement: Supplementary file 6 [file Image6.tiff]

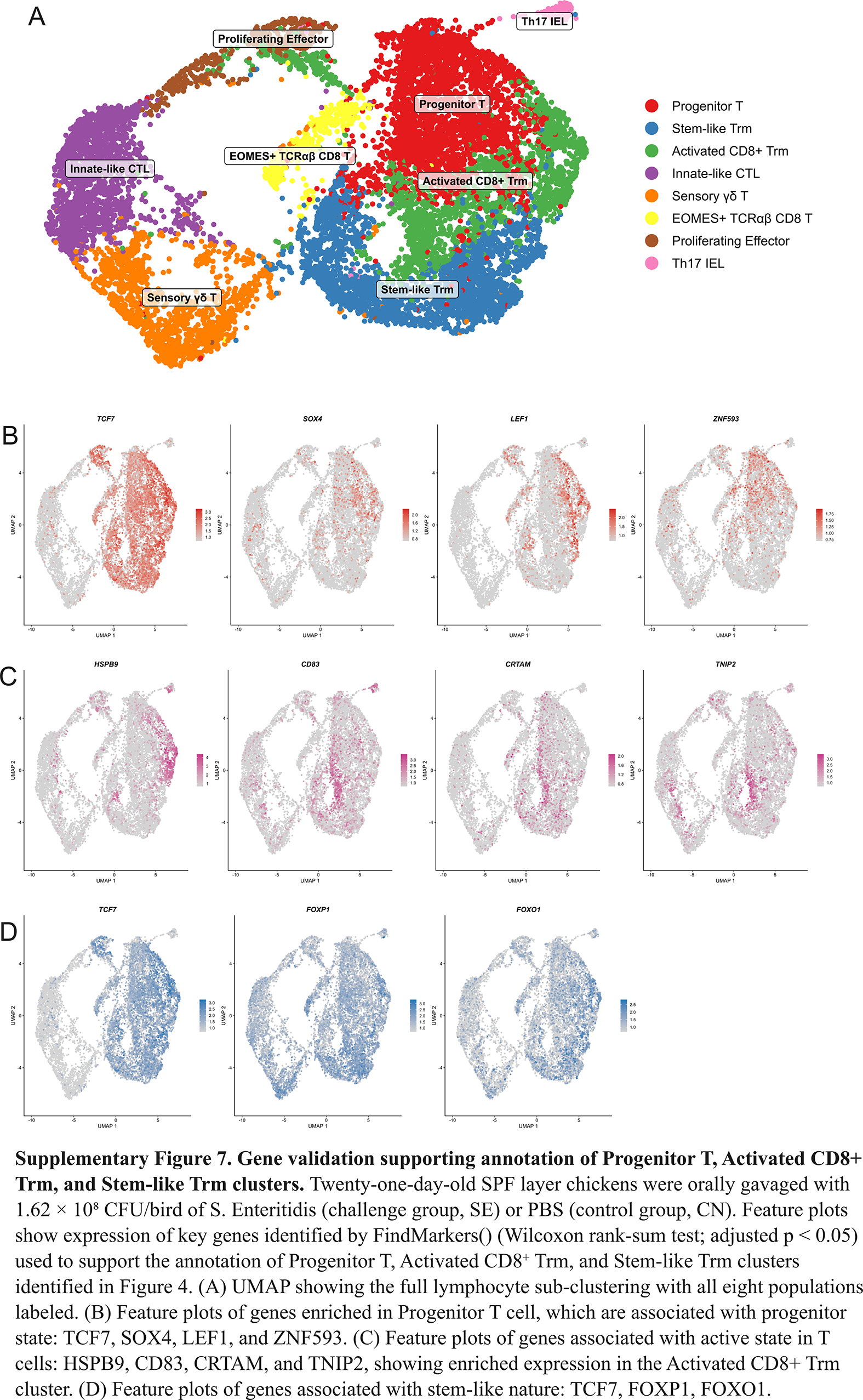

Supplement: Supplementary file 7 [file Image7.tiff]

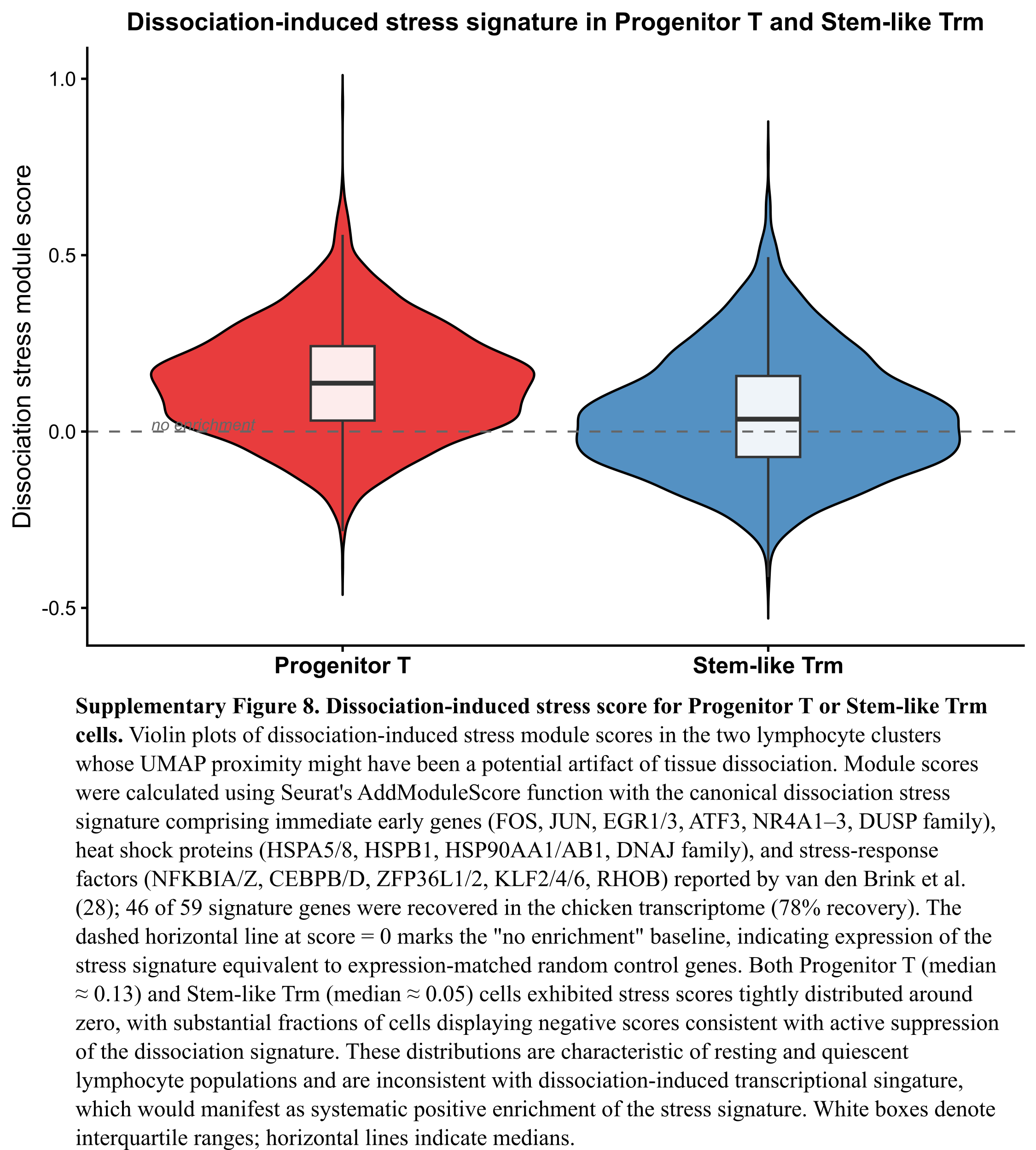

Supplement: Supplementary file 8 [file Image8.tiff]

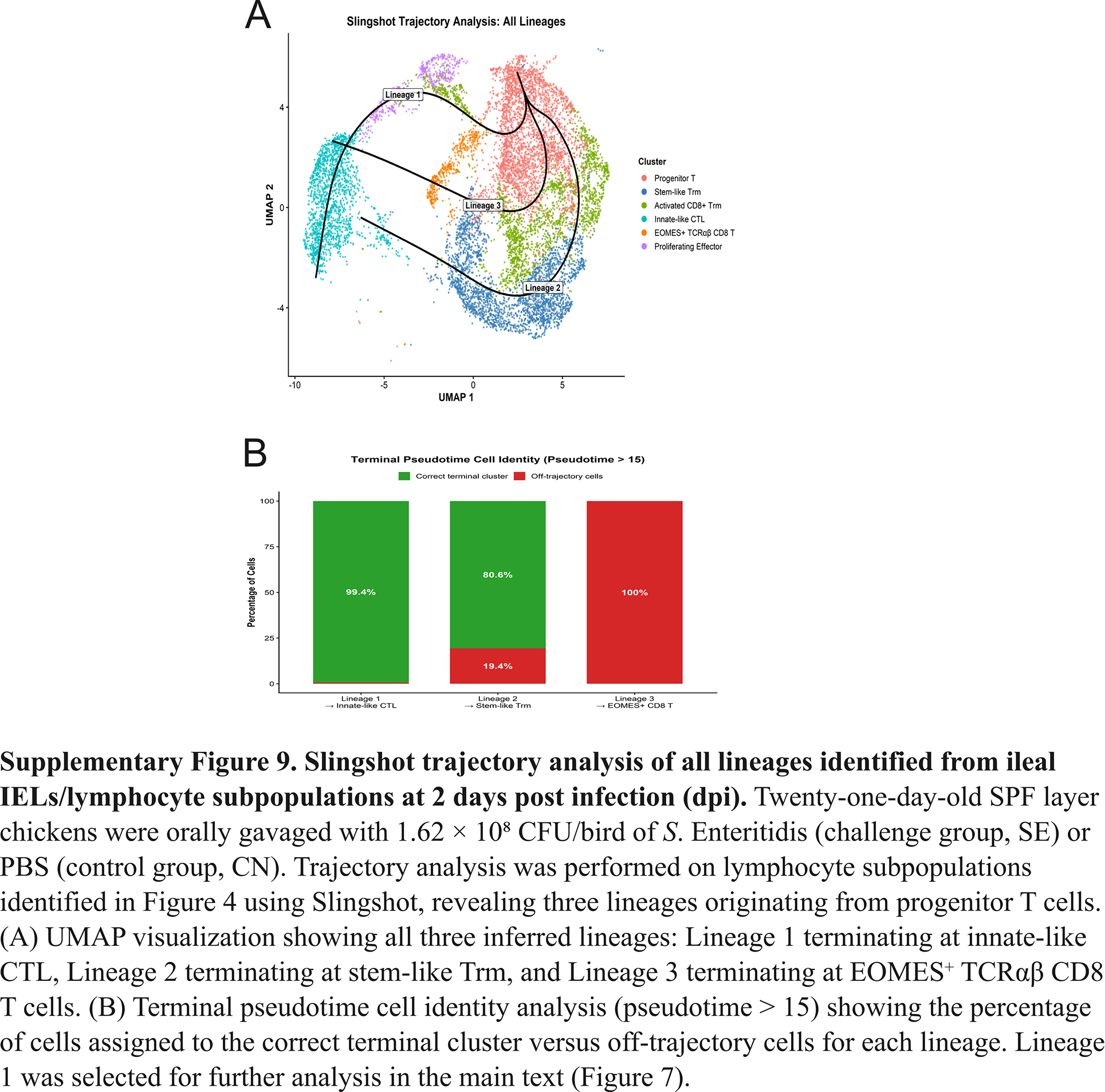

Supplement: Supplementary file 9 [file Image9.tiff]
